# A tablet-based attentional cognitive bias modification intervention for positive emotion processing in major depressive disorder: a single-blind randomised controlled two-centre trial

**DOI:** 10.1016/j.ebiom.2026.106453

**Published:** 2026-09-01

**Authors:** Zoe Katharina Kratochwil, Jessica Kesik, Duygu Keskin-Gökcelli, Katrin Sakreida, Norbert Scherbaum, Bernhard Wolfram Müller, Thomas Frodl

**Affiliations:** a LVR-University Hospital Essen, Department of Psychiatry and Psychotherapy, Faculty of Medicine, University of Duisburg-Essen, Virchowstraße 174, Essen, 45147, Germany; b Department of Psychology, University of Wuppertal, Gaußstraße 20, Wuppertal, 42119, Germany; c Hospital of Duisburg-Essen, Centre for Translational Neuro and Behavioural Sciences (C-TNBS), Medical Faculty University, Hufelandstraße 55, Essen, 45147, Germany; d Department of Psychiatry, Psychotherapy and Psychosomatics, RWTH Aachen University, Pauwelsstraße 30, Aachen, 52074, Germany; e German Centre for Mental Health (DZPG), partner site Halle-Jena-Magdeburg, Philosophenweg 3, Jena, 07742, Germany

**Keywords:** Cognitive bias modification (CBM), Attention bias, Psychiatry, Emotion-processing task, Major depressive disorder, Early posterior negativity

## Abstract

**Background:**

Patients with Major Depressive Disorder (MDD) exhibit biased information processing. This study investigates the clinical and neural efficacy of a cognitive bias modification (CBM) training in patients and healthy control (HCS), fostering processing of positive emotion stimuli.

**Methods:**

In a single-blind randomised controlled trial (DRKS00029756), MDD outpatients and HCS, aged 18–60 years, were randomised to positivity training (PT) or control training (CT). Randomisation was carried out by study assistants, blinding researchers. Participants underwent 10 tablet-based dot-probe sessions over 14 days. PT implicitly redirected attention toward positive stimuli to improve depressive symptoms. Primary outcome was pre and post-training EEG-derived Early Posterior Negativity (EPN), investigating neural sensitivity towards positive stimuli. Secondary clinical outcomes included changes in depression symptoms.

**Findings:**

From February 2023 to October 2024, 240 participants were included (119 MDD; 121 HCS). 62 MDD and 61 HCS underwent PT; 57 MDD and 60 HCS received CT. In MDD, EPN to positive high arousal stimuli revealed an interaction between time (pre/post) and training (PT/CT), F(1,97) = 5.017, *p* < 0.028, η^2^ = 0.049. The between-treatment difference was 1.65 μV, 95% CI [0.19, 3.11]. EPN amplitudes shifted towards negativity in PT and positivity in CT. Depression symptom decreased from pre to post and from pre to follow-up. With respect to adverse events, one patient in the control training was regularly admitted to hospital.

**Interpretation:**

CBM positivity training was associated with neural changes in MDD, supporting its potential to target pre-attentive positive emotion processing. Further EEG follow-up and higher stimulus contrasts in the training conditions may clarify clinical relevance.

**Funding:**

Funded by 10.13039/501100001659DFG (ID: 507720021).

**Research in context**

**Evidence before this study**

We reviewed clinical trials reporting on the effectiveness of cognitive bias modification (CBM) interventions targeting the negative attentional bias in adult patients with Major Depressive Disorder (MDD) from November 1st, 2022, until December 15th, 2024. Research articles were searched via PubMed, using the keywords ‘cognitive bias modification’, ‘attention’ and ‘depression ‘, and ‘adults’. Reviewed articles were published in English and available in full text. The search yielded 46 results, including 24 clinical studies, 23 randomised controlled trials, three systematic reviews, and two meta-analyses, though 12 randomised controlled trials and 13 clinical trials were excluded from revision, since the interventions did not target patients with MDD. Only one randomised controlled trial was found, using a dot probe task in clinically depressed patients, comparing different training conditions. Vrijsen and colleagues, however, investigated cognitive bias modification in an inpatient setting on computers and found clinical improvements to be greatest for patients receiving active dot-probe training for cognitive bias modification. This prompted the exploration of using study tablets for the same training task and evaluating its effectiveness in outpatients, aiming to offer a time- and cost-effective add-on treatment, particularly in rural areas.

To gain a more comprehensive understanding of the training’s effectiveness, Early Posterior Negativity (EPN), reflecting rapid, automatic attentionally engagement with emotionally salient stimuli, was integrated as a neurophysiological measure, aiming to investigate possible early neural markers of training efficacy. Currently there is a lack of integrating EPN when researching cognitive bias modification trainings in depressed individuals.

**Added value of this study**

The present study was a single-blind, randomised controlled trial over 6 weeks, containing a two-week, tablet-based cognitive training intervention for patients with MDD as well as healthy controls (HCS). After a post-training assessment, we included a four-week follow-up period to investigate the potential longevity of a cognitive training effect on depression symptoms. The primary outcome EPN was integrated by measuring two EEGs (pre vs. post training) to investigate possible changes in neural sensitivity towards positive material after training completion. In terms of neural changes, active CBM training was associated with changes in EPN only in patients, wherein EPN amplitudes of patients shifted towards negativity in positivity training (PT) from pre- to post-investigation, while EPN amplitudes of patients in control training (CT) shifted towards positivity. Clinically, we found an overall improvement in all training groups, for MDD and HCS subjects alike (though HCS showed a smaller improvement). We also investigated potential effects of medication status, anxiety, sex, age, and childhood trauma on training effectiveness, though none of these factors significantly influenced changes in depression in patients of either training group. Childhood trauma was associated with effectiveness, however, only on HCS in the control training.

**Implications of all the available evidence**

The reported findings are promising, particularly concerning the neural changes, marked by EPN. CBM training also improved depression symptoms in both active and control training groups, implying either a general positive effect of cognitive training, independent from its condition, or positive external influences caused by the study participation (i.e., regular on-site contact, structured daily life, etc.). Research on the clinical effectiveness of CBM trainings in clinically depressed patients remains inconclusive because of the persisting heterogeneity of study designs (i.e., training tasks, training frequency, training setting, included patient populations, etc.). The present study included a larger cohort than most other studies researching cognitive bias modification in depressed patients, as well as a healthy control group. Further research with a comparable study design is needed to investigate these ideas.

## Introduction

According to the World Health Organisation^1^ Major Depressive Disorder (MDD) remains the most prevalent psychiatric disorder. As the leading cause of lost work productivity, disability, long-term sick leave, and early retirement in the EU, MDD exerts a substantial socioeconomic burden. Despite advances in treatment, achieving effective and sustained remission remains a major clinical challenge. Medication and cognitive therapy are effective; however, 30% of patients (i. e., 9 million patients in Europe alone) do not respond to standard treatments even after multiple treatment steps.^2^ The main symptoms of MDD, entailing depressed mood and loss of interest and pleasure, are highly associated with conceptualised negative beliefs about the self, the world, and the future.^3^ Beck’s cognitive model of depression builds upon altered physiological, attention, and memory functioning related to preferential processing of negative information. Negative schemata influence information processing by orienting salience to negative events and decreasing attention to positive events.^4^ However, altered processing of positive information observed in MDD reflects not merely a shift towards mood-congruent negative biases, but rather an active inhibition of rewarding stimuli through devaluation of positive inputs.^5^, ^6^, ^7^ In their tripartite model of depression and anxiety, Clark and Watson^8^ formulated low positive affect as an important dimension characterising depression.

Based on these models, several experimental techniques have been developed to alter maladaptive cognitive biases and restore distorted information processing.^9^ These methods are commonly referred to as Cognitive Bias Modification (CBM). Studies on CBM training in MDD primarily aim to address negatively biased interpretation, or attention. Previous studies reported improvements in the interpretation bias and positive acceptance of a smartphone-based CBM among participants,^10^ as well as increased activation of the striatum and medial prefrontal cortex following active approach positivity training.^11^ Recent studies and meta-analyses suggest that CBM training focusing on biased interpretation or attention is most effective, though results are mixed, due to heterogeneity in training tasks and frequency.^12^, ^13^, ^14^, ^15^, ^16^, ^17^ One of the most widely used tasks in attentional CBM training is the Dot Probe task (DPT). A specific variation employs positive images or words to shift attention toward positive information, aiming to reduce the negative attentional bias.^13^ Previous CBM studies reported no (serious) adverse events and partial clinical improvements, suggesting low risk and potential benefits for participants.

Vrijsen et al.^18^ investigated the effects of a positivity-increasing attention DPT, and a positivity-approach training (AAT) compared to a control training (CT) in a blinded randomised controlled design in patients with MDD and healthy controls (HCS). Among 139 inpatients who received four sessions of DPT or AAT, alongside treatment as usual, clinician-rated symptom severity decreased significantly more in patients in active DPT or AAT, compared to the control condition. Previous studies focused predominantly on clinical outcomes, with few entailing (neuro-)physiological measurements such as fMRI, eye tracking, or EEG.^19^^,^^20^ Two EEG studies have explored event-related potentials following CBM focusing on visual mismatch negativity (P100) and P200 amplitudes.^21^^,^^22^ To better understand early-stage emotional processing in MDD, event-related potentials (ERPs) offer a valuable neurophysiological perspective. The Early Posterior Negativity (EPN), an ERP component occurring approximately 150–300 ms after stimulus onset, reflects rapid, automatic attentional engagement with emotionally salient stimuli and is largely independent of task demands.^23^, ^24^, ^25^, ^26^ The EPN is inherently a negative-going wave relative to the average baseline activity or a neutral stimulus condition.^27^ Its negativity reflects increased neural activity in brain regions involved in visual processing, and has been linked to enhanced attention to emotional stimuli.^28^ In patients with MDD, EPN responses were attenuated and delayed in response to positive emotional content, indicating impaired early affective processing.^29^ In a previous study investigating a non-pharmacological intervention in oncology patients, we found the EPN to be sensitive to positive valence stimuli.^30^ The aim of the present randomised controlled clinical trial was to investigate the effect of a tablet-delivered attention bias modification intervention on EPN to positive valence stimuli as a primary outcome parameter of automated emotion processing to implicitly improve positive emotion processing in MDD and in HCS. This mobile format enhances the feasibility of dissemination in primary care and rural settings, increasing its potential for wider clinical application. By including both clinical and healthy samples, we aim to investigate MDD-specific neuronal processing of positive emotional information, and to examine the training’s effectiveness considering symptom improvements over time.

## Methods

### Oversight

This single-blinded, randomised, placebo-controlled trial primarily aimed to assess neurophysiological effects of CBM using EPN measures. Funded by the DFG (507720021), the study was registered in the German Clinical Trials Registry (DRKS) and the WHO International Clinical Trials Registry (ICTRP; No. DRKS00029756) and was conducted at the RWTH University Hospital in Aachen and the LVR University Hospital in Essen with home-based training on tablets. Participants received a reimbursement of 100 Euros for their time and effort during the visits in accordance with standard practice in German clinical research. The study was conducted in compliance with the study protocol (Version 1, dated 11 February 2022), which represents the final approved version at the time of study initiation. No protocol amendments affected outcomes, analyses, or interpretation of the data. The primary research question, outcome measures, and statistical analysis plan remained unchanged.

### Sample size and power calculation

Sample size estimation was based on preliminary data and established parameters reported in the EPN literature. Group-level mean EPN amplitudes for high-arousal stimuli typically fall within or slightly above the 1–2 μV range, with reported means such as 1.58–3.16 μV depending on stimulus type and analysis window.^23^^,^^31^, ^32^, ^33^ In our preliminary data (Fink et al., 2024), we found EPN amplitude-related evoked potential changes associated with emotion conditions (−1.46 μV, SD 1.7 μV). For power calculation, utilising a continuous calculation approach, we use the high positive condition, and for effect size determination, we assume a small, non-specific effect in the clinical control condition training group of −0.2 μV (SD = 1.7). In the clinical positivity training group, we assume an additional training effect of 1.0 μV, resulting in a pre/post positivity training EPN change of −1.2 μV (SD = 1.7) among patients in the positivity training. Using G∗Power (V3.1.9.7), this results in a moderate effect size of Cohen’s *d* = 0.58.^34^ Given alpha = 0.05 and 1–beta = 0.8, in a two-tailed t-test using treatment related (post-pre) difference scores in two independent groups (patients with positivity training (−1.2 μV) vs. patients with control training (−0.2 μV)) a total sample size of 94 (=2 × 47) subjects is needed to reach a critical t of 1.99 as indicated by G∗Power (V3.1.9.7). Considering dropouts due to technical problems and patient compliance of about 20%, an initial sample size of 120 patients is sufficient to detect treatment-related changes in emotion processing as indicated by the EPN component in the processing of high-arousal positive valence stimuli. No formal sensitivity analysis was performed. Instead, conservative assumptions, including a larger standard deviation and minimal clinically important effects, were used to ensure a robust sample size of 120 patients.

### Design

This is a two-centre, single-blind, randomised controlled clinical trial with two arms (active CBM training vs. control training) in patients with MDD and HCS.

#### Randomisation

Participants were randomised in blocks of ten MDD and ten HCS per site (Aachen/Essen) using pre-generated block randomisation lists from ‘Research Randomizer’ (Version 4.0;^35^). Block randomisation ensured balanced group sizes across sites and diagnostic groups and aimed to match HCS to patients for age and sex.

#### Allocation concealment

Group assignments were kept in sealed, opaque envelopes and only revealed after participants completed baseline assessments. The envelopes were handled by blinded assistants not involved in outcome assessment, ensuring proper allocation concealment.

#### Safety monitoring and interim analyses

Please see Appendix B-2 for details.

#### Blinding

Outcome assessors and data analysts were blinded to group assignment throughout the trial. Participants were informed they would receive one of two training conditions (CT or PT) but not about their training assignment. Both interventions used the same task and stimuli, but targets followed positive images in 100% of PT vs. 50% of CT trials.

### CBM training and task

Participants were randomly assigned to PT or CT (Allocation ratio: 50% each; see Supplemental Fig. S1, Tables 1 and 2 on sociodemographic data) and completed ten 15−minute sessions of dot-probe CBM task over 14 days on a Samsung Galaxy Tab A8 (Android 11). The intervention frequency was based on prior evidence suggesting clinically meaningful effects for a multi-session protocol of approximately 6–10 sessions.^36^ Earlier depression studies used shorter protocols (e. g., four sessions),^18^ whereas related CBM research suggests more robust clinical effects for multi-session protocols of 4–12 sessions.^18^^,^^37^^,^^38^ Learning-curve analyses indicate improvements plateau around 6 sessions, although additional training may provide incremental benefits.^36^^,^^39^^,^^40^ Taken together, the evidence suggests an effective range of 6–10 sessions. Accordingly, 10 sessions over 14 days were chosen as a feasible, empirically supported protocol providing sufficient intervention intensity to detect potential neurophysiological and clinical effects. The DPT (Presentation app, Neurobehavioral Systems Inc.) used 100 positive and 100 neutral IAPS images (see Supplemental Table A1 on means and standard deviation across arousal, valence, and dominance levels). High-arousal positive images were selected from the IAPS based on normative valence and arousal ratings to ensure clearly positive, high-arousal content with a balanced distribution. The final set comprised high-valence, high-arousal positive scenes (e.g., affiliative interactions, social reward, pleasant nature scenes) without extremely explicit material.

**Table 1:** Sociodemographic characteristics of patients with major depressive disorder (MDD) across training conditions (*N* = 119).

	MDD CT (n = 57)	MDD PT (n = 62)	Overall MDD (n = 119)
Age			
M [SD]	34.79 [12.02]	37.51 [13.52]	36.18 [12.83]
Median [Q1; Q3]	33.00 [24.00; 43.25]	35.00 [24.00; 51.50]	33.00 [24.00; 50.00]
Sex			
Men/Women	25/32	19/43	44/75
Centre			
Aachen/Essen	30/27	30/32	60/59
Baseline MADRS			
M [SD]	15.086 [9.28]	13.767 [9.36]	14.42 [9.27]
Median [Q1; Q3]	14.50 [8.00; 20.25]	12.00 [8.00; 20.00]	14.00 [8.00; 20.00]
Baseline HAMD-21			
M [SD]	12.724 [6.95]	11.567 [7.01]	12.14 [6.95]
Median [Q1; Q3]	13.50 [7.75; 17.00]	12.00 [7.00; 16.00]	13.00 [7.00; 16.00]
Baseline BDI-II			
M [SD]	24.776 [12.49]	22.016 [12.49]	23.36 [12.52]
Median [Q1; Q3]	25.50 [14.75; 32.25]	19.00 [12.00; 31.00]	23.00 [14.00; 32.00]
Baseline STAI-X2	56.29 [11.07]	54.97 [11.07]	55.61 [11.05]
Median [Q1; Q3]	56.00 [45.00; 62.25]	51.00 [43.00; 62.50]	55.00 [44.00; 63.00]
Concomitant treatments			
Yes/No	42/15	48/14	90/29
Past psychiatric treatments			
Yes/No	44/13	49/13	93/26

MDD = major depressive disorder subjects; CT = control training; PT = positivitye training. Age is reported as mean (M) and standard deviation (SD). BDI-II = Beck Depression Inventory–II; HAMD-21 = 21-item Hamilton Depression Rating Scale; MADRS = Montgomery–Åsberg Depression Rating Scale; STAI-X2 = State- Trait Anxiety Inventory—State Subscale. Values are means (M) and standard deviations (SD).

**Table 2:** Sociodemographic characteristics of healthy control subjects (HCS) across training conditions (*N* = 121).

	HCS CT (n = 60)	HCS PT (n = 61)	Overall HCS (n = 121)
Age			
M [SD]	32.881 [11.02]	34.52 [14.59]	33.72 [12.95]
Median [Q1; Q3]	29.00 [23.00; 41.00]	29.50 [24.00; 44.00]	29.00 [24.00; 42.00]
Sex			
Men/Women	20/40	15/46	35/86
Centre			
Aachen/Essen	30/30	30/31	60/61
Baseline MADRS			
M [SD]	0.690 [1.97]	0.610 [2.00]	0.65 [1.94]
Median [Q1; Q3]	0.00 [0.00; 1.00]	0.00 [0.00; 0.00]	0.00 [0.00; 0.00]
Baseline HAMD-21			
M [SD]	0.914 [1.62]	0.847 [1.65]	0.88 [1.60]
Median [Q1; Q3]	0.00 [0.00; 2.00]	0.00 [0.00; 1.00]	0.00 [0.00; 1.00]
Baseline BDI-II			
M [SD]	3.465 [4.47]	3.565 [4.46]	3.52 [4.45]
Median [Q1; Q3]	2.00 [0.00; 6.00]	2.00 [0.00; 6.00]	2.00 [0.00; 6.00]
Baseline STAI-X2	33.80 [9.84]	33.03 [9.84]	33.41 [9.81]
Median [Q1; Q3]	33.00 [29.00; 40.00]	34.00 [28.75; 44.25]	33.00 [29.00; 42.00]
Concomitant treatments			
Yes/No	0/60	2/59	2/119
Past psychiatric treatments			
Yes/No	1/59	6/55	7/114

HCS = healthy control subjects; MDD = major depressive disorder subjects; CT = control training; PT = positivity training. Age is reported as mean (M) and standard deviation (SD). BDI-II = Beck Depression Inventory–II; HAMD-21 = 21-item Hamilton Depression Rating Scale; MADRS = Montgomery–Åsberg Depression Rating Scale; STAI-X2 = State- Trait Anxiety Inventory—State Subscale. Values are means (M) and standard deviations (SD).

### Emotion processing task (EPT)

EPT was used to assess neural responses to emotional stimuli, presenting 800 IAPS images in randomised order. Analyses focused on high-arousal positive stimuli (n = 80; 10% of total). Each image was shown for 500 ms, followed by a 2.5 s stimulus onset asynchrony (SOA) and a 540 ms fixation cross. Images were framed in red or blue; participants responded to red-framed stimuli via mouse button press to ensure attentional control,^41^ which was true for all emotion stimuli.

### Participants

**Fig. 1:**
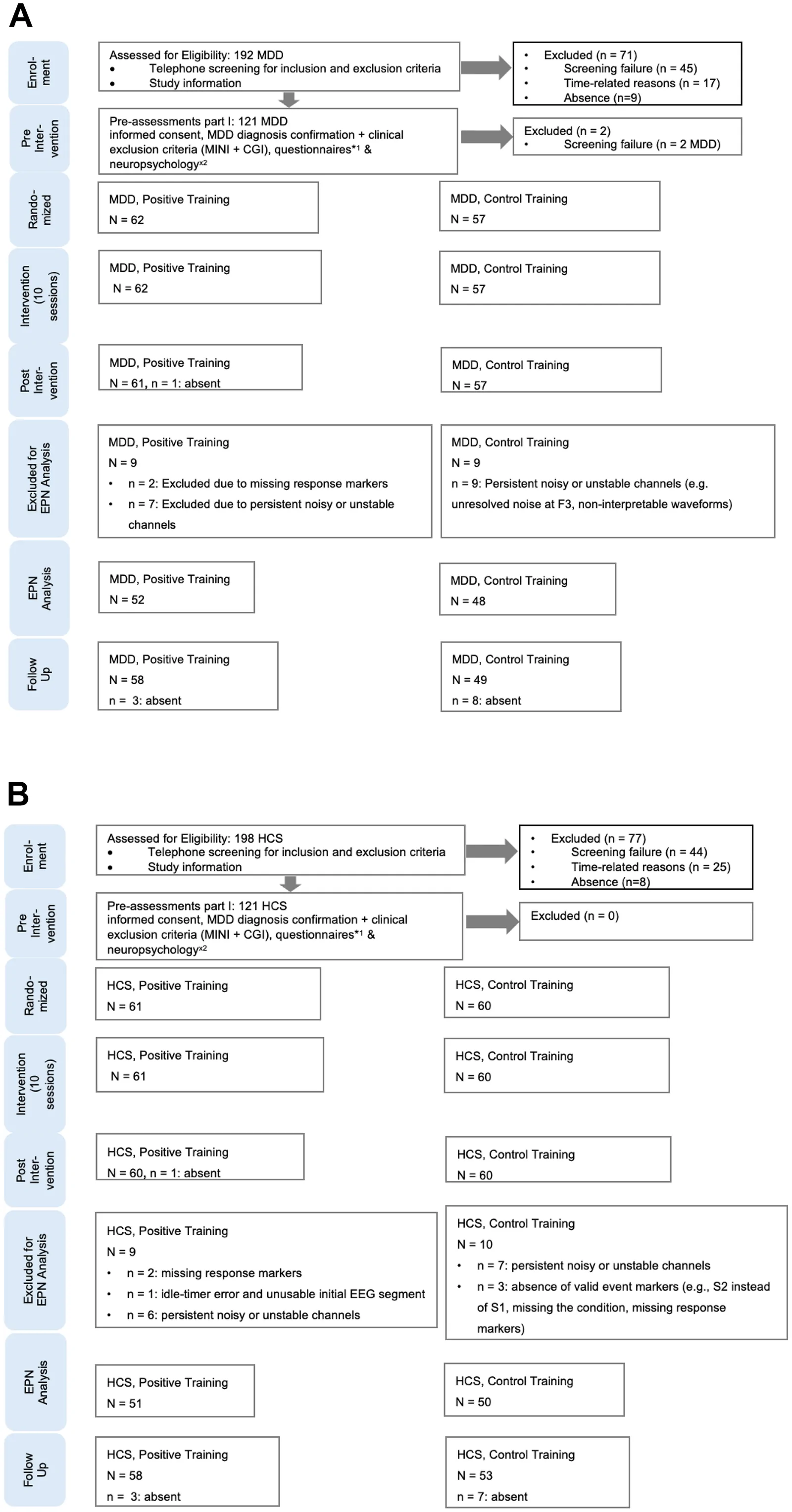
Consort flow diagram of included subjects in the Major Depressive Disorder (MDD) sample **(A)** and Healthy Control subjects (HCS) sample **(B)**.

390 participants were assessed for eligibility. A total of 242 participants completed screening; 240 met eligibility criteria and were included in the pre-assessment (Fig. 1). Patients, aged between 18 and 60 years, required a primary diagnosis of MDD (verified by the Mini-International Neuropsychiatric Interview during pre–assessment). Sex was self-reported during screening and recorded as male or female; gender identity was not separately assessed. Concurrent psychotropic medication in patients was permitted, provided that the type of medication (see Supplemental Table A3 for numbers on antidepressant intake) and dosage had persisted for 4 weeks before randomisation (6 weeks in case of Fluoxetine). Concurrent psychotropic medication and dosage were documented during pre–assessments. Furthermore, patients were explicitly asked to remain stable in their medication and dosage until participation ended. Exclusion criteria (for both groups) included a history of psychosis or mania, substance abuse (except nicotine), neurological disorders, pregnancy, untreated hyperthyroidism, diabetes (HbA1c > 7.5), and stroke. For inclusion, HCS had to be aged 18–60 years and were not permitted to meet any DSM-V criteria for a current or past psychiatric disorder as assessed by the M.I.N.I, see Tables 3 and 4 for depression severity from MADRS, HAMD-21, and BDI-II over timepoints.

**Table 3:** Table of primary (EPN) and secondary (depression scores) outcome measures at post-training and follow-up for patients with Major Depression.

EPN in μV						
Time point	Mean CT (SD)	Mean PT (SD)	Point estimate (CT–PT)	95% CI for difference	*p*-value	Cohen’s *d*
Post-training	3.70 (2.85)	2.90 (3.53)	0.80	[−0.48, 2.08]	0.22	0.25

MADRS						
Time point	Mean CT (SD)	Mean PT (SD)	Point estimate (CT–PT)	95% CI for difference	*p*-value	Cohen’s *d*
Post-training	11.50 (9.43)	11.60 (9.52)	−0.10	[−3.56, 3.36]	0.95	−0.01
Follow-up	11.62 (9.81)	10.65 (9.48)	0.97	[−2.55, 4.49]	0.59	0.10

HAMD-21						
Time point	Mean CT (SD)	Mean PT (SD)	Point estimate (CT–PT)	95% CI for difference	*p*-value	Cohen’s *d*
Post-training	9.21 (6.64)	8.77 (8.57)	0.44	[−2.36, 3.24]	0.76	0.06
Follow-up	9.10 (7.77)	8.75 (6.98)	0.32	[−2.37, 3.01]	0.82	0.04

BDI-II						
Time point	Mean CT (SD)	Mean PT (SD)	Point estimate (CT–PT)	95% CI for difference	*p*-value	Cohen’s *d*
Post-training	19.26 (12.21)	17.39 (13.71)	1.87	[−2.86, 6.59]	0.44	0.14
Follow-up	17.83 (13.52)	17.64 (14.03)	0.19	[−4.82, 5.19]	0.94	0.01

Only the primary time point (post-training) was reported for EPN, since no EEG was conducted for follow-up.

**Table 4:** Table of primary (EPN) and secondary (depression scores) outcome measures at post-training and follow-up for healthy control subjects.

EPN in μV						
time point	Mean CT (SD)	Mean PT (SD)	Point estimate (CT–PT)	95% CI for difference	*p*-value	Cohen’s *d*
Post-training	3.06 (3.13)	3.49 (3.40)	−0.43	[−1.72, 0.86]	0.51	−0.13

MADRS						
Time point	Mean CT (SD)	Mean PT (SD)	Point estimate (CT–PT)	95% CI for difference	*p*-value	Cohen’s *d*
Post-training	0.98 (2.28)	0.68 (2.06)	0.30	[−0.49, 1.10]	0.45	0.14
Follow-up	1.10 (2.82)	0.95 (2.25)	0.14	[−0.80, 1.07]	0.77	0.05

HAMD-21						
Time point	Mean CT (SD)	Mean PT (SD)	Point estimate (CT–PT)	95% CI for difference	*p*-value	Cohen’s *d*
Post-training	0.95 (2.18)	0.68 (1.78)	0.27	[−0.46, 1.00]	0.46	0.14
Follow-up	1.19 (3.18)	1.27 (2.47)	−0.08	[−1.12, 0.96]	0.88	−0.03

BDI-II						
Time point	Mean CT (SD)	Mean PT (SD)	Point estimate (CT–PT)	95% CI for difference	*p*-value	Cohen’s *d*
Post-training	1.75 (3.05)	2.65 (5.89)	−0.90	[−2.60, 0.80]	0.30	−0.19
Follow-up	1.58 (2.93)	2.27 (4.30)	−0.70	[−2.03, 0.62]	0.30	−0.19

Only the primary time point (post-training) was reported for EPN, since no EEG was conducted for follow-up.

### EEG recording

**Fig. 2:**
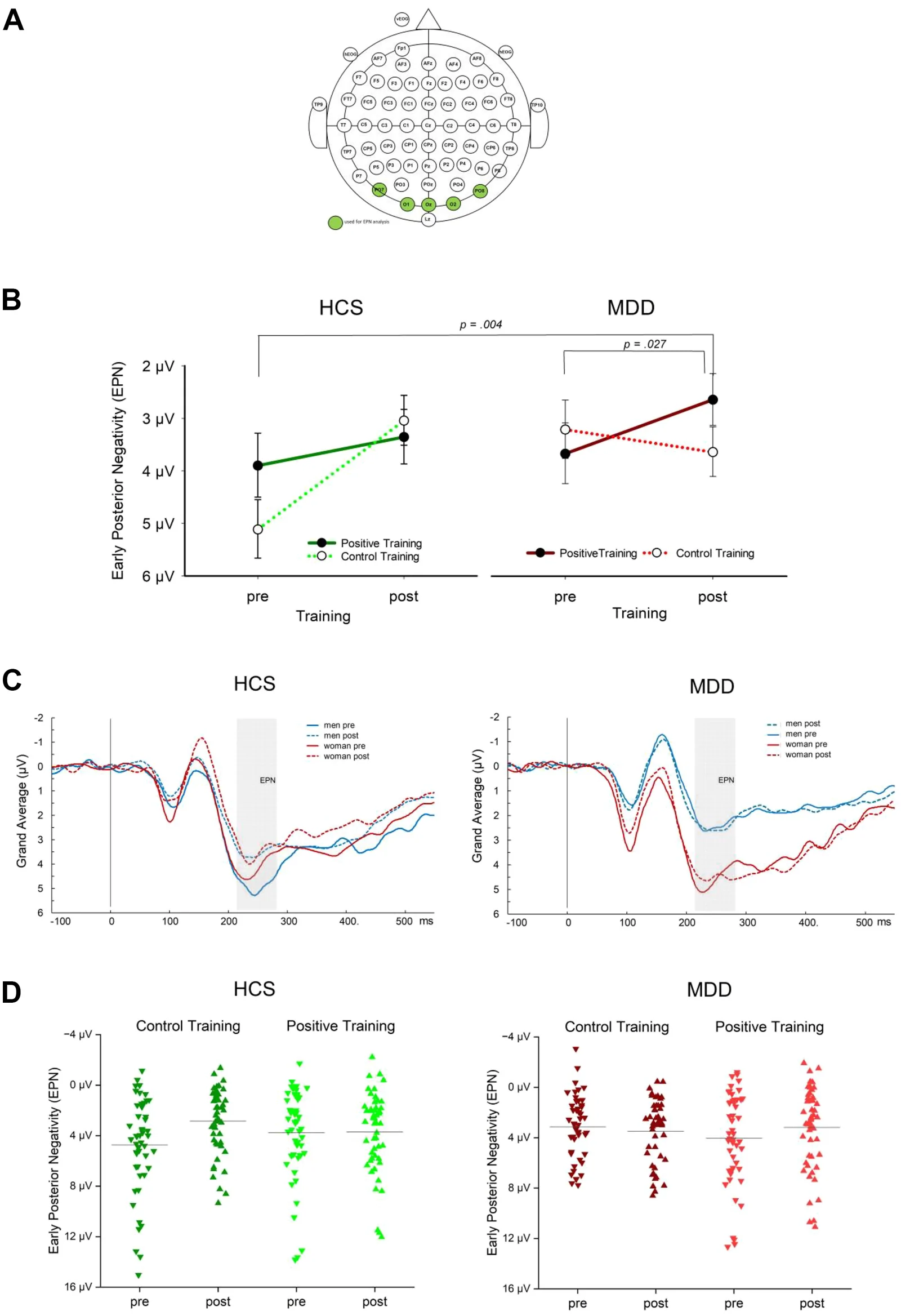
**(A)** Positions of the 64 electrodes, including their numbers and designations, are based on the internationally recognised 10–20 system and used Electrodes for EPN data analysis (marked in green) (Source: Demin et al., 2016). **(B)** Early Posterior Negativity (EPN) mean ( ± 1 SE) data in patients (MDD) and controls (HCS). Pre and post training data for the positivity training (PT) and the control training groups (CT). The primary outcome EPN in MDD (right) resulted in a significant time (pre/post) ∗ training (PT/CT) interaction (right). A secondary analysis comparing time (pre/post), intervention (CT/PT) and subject group (MDD/HCT) revealed a significant three-way interaction of CT and PT on EPN in HCT and MDD groups over time. EEG convention negative up. **(C)** Grand average waveforms of early posterior negativity potential (EPN) in microvolt (μV) for men vs. women at pre and post measurement for positive valence high arousal stimuli in HCS (left) and MDD (left) right. Grand average data for men (blue) and woman (red), pre (straight line) and post intervention (dotted line) from −100 ms prior to 600 ms post stimulus onset (x-axis). A significant sex difference was found in MDD with men showing more negative EPNs compared to women, F(1,97) = 7.845, *p* = 0.006, with no other main or interaction effects. No significant effects of sex were found in controls. EEG convention negative up. **(D)** Dot density plot of Early Posterior Negativity (EPN) amplitudes before (pre) and after (post) training in HCS (left) and in MDD (right) for pre and post assessments in the CT and PT groups. Dots represent individual subjects EPN amplitudes. Dark lines indicate group means. EEG convention negative up. Note. For (C) the x-axis represents latency in milliseconds (ms) and the y-axis represents EPN amplitude in microvolt (μV). For (B) and (D) the x-axis represents time points (pre, post and follow-up (D only)) and the y-axis represents EPN amplitude in microvolt (μV).

Continuous EEG was recorded in two sessions, two weeks apart, using a 64-channel actiCAP system with active Ag/AgCl electrodes (Brain Products GmbH, Germany), at a sampling rate of 1000 Hz via Brain Vision Recorder (v1.10) within a frequency range of 0.01 Hz–250 Hz. Electrodes were placed following the international 10/20 system, with FPz as ground and FCz as reference. TP9 and TP10 (earlobes) served as offline re-reference. Additional electrodes were placed beside the left and right and below the right eye to monitor ocular activity. Stimuli were presented on a 24−inch monitor (1920 × 1200 px) at 2−metres distance (visual angle ∼6.3°), mirrored on a screen in an adjacent room. Offline pre-processing was performed in Brain Vision Analyser (v2.3.0). Data were band-pass filtered (0.03–30 Hz, 8th-order Butterworth), then visually inspected for artefacts. Electrodes that showed visible deviations from surrounding electrodes were excluded and recalculated by pooling data from the four surrounding electrodes. Afterwards, the data were further cleaned using semi-automatic ICA (restricted infomax algorithm) trained on 360 s of artifact-free data.^42^ ICA components arising from vertical or horizontal eye movement were identified using the sum of squared correlations with vertical and horizontal ocular activity. The total value to delete was set to 30%. EEG data were segmented (100 ms pre-stimulus − 1000 ms post-stimulus), baseline-corrected (−100 ms to 0 ms), and time-locked to stimulus onset. Only trials with correct responses were included. Single-subject averages were computed to identify ERP components. The EPN was computed as mean voltage in the time range of 220 ms to 280 ms following stimulus onset at parieto-occipital electrodes (PO7, O1, O2, Oz, PO8; Fig. 2A).

### Assessments

Participants attended four on-site appointments. The first assessment (one day before training (PT/CT) start) included diagnostic and psychosocial evaluation. MDD diagnosis (or its absence in HCS) was confirmed via the M.I.N.I. Clinical raters administered the MADRS,^43^ the Hamilton Depression Rating Scale (HAMD-21)^44^; and the Clinical Global Impression Scale (CGI)^45^; self-report measures included the Cumulative Illness Rating Scale (CIRS),^46^^,^^47^ Work and Social Adjustment Scale (WSAS),^48^ Child Trauma Questionnaire (CTQ),^49^ the Barratt Impulsiveness Scale (BIS-11),^50^ and State-Trait Anxiety Inventory (STAI).^51^; Demographics and neuropsychological (memory, executive functioning, attention, processing speed) were also collected. The second assessment (one day post-screening) included EEG and repeated symptom measures (BDI-II, MADRS, HAMD-21, STAI-X1/X2); mean post-pre interval: 17.22 days. Participants completed ten training sessions (mean: MDD: 9.09 ± 0.256; HCS: 9.65 ± 1.660; (t(189.46) = 1.860, *p* < 0.001). Final assessment occurred four weeks post-intervention (means: pre-follow-up: 49.65 days; post-follow-up: 32.35 days). At final assessment, MADRS and HAMD-21 were administered, and self-report measures on depression (BDI-II) and state-trait anxiety were conducted one final time. At baseline, 72 patients with MDD were on antidepressant medication; 47 were medication-free (see Supplemental Tables A2 and A3 for specific numbers for all subgroups).

### Safety

No formal benefit or harm assessment was conducted. Participants remained in contact with the study team throughout participation and could report adverse events or concerns at any visit.

### Ethics

The study was approved by the Ethics committees of the Universities of Aachen (EK-109-22) and Essen (22-10640-KOBOb) and was conducted in accordance with the ethical principles outlined in the 2024 Declaration of Helsinki. All participants provided written informed consent prior to participation.

### Statistics

#### Data management and handling

Clinical and experimental data were collected using paper-based forms and transferred to IBM SPSS Statistics by trained personnel, with independent cross-checking to ensure accuracy. Participants were pseudonymised using identification codes, with the key stored separately in a secure location. Electronic data were stored on password-protected offline computers, accessible only to authorised study personnel and managed according to institutional data protection policies. Analyses were performed using Statistical Package for the Social Sciences (SPSS) version 30 (IBM Corp). Degrees of freedom were estimated via Satterthwaite approximation. To model within-patient errors, an unstructured covariance matrix was assumed. For all analyses, the significance level was set to two-sided *α* = 0.05. 95%, two-sided confidence intervals are reported. As no predefined benefit or harm outcomes were implemented, no formal group comparisons for harms were conducted. One anticipated adverse event occurred in a CT patient who entered inpatient treatment during study participation.

#### EPN data

##### Primary outcome and confirmatory analysis

The primary endpoint was assessed with two-tailed *t*-tests for independent groups on difference scores on the EPN in the high arousal positive valence condition between the patient experimental and patient control group and between the patient experimental and the control experimental positivity training group. Given potential influences of age and sex, depression severity and medication intake as exploratory variables, all EPN analyses were also confirmed using mixed-design ANCOVAs to avoid alpha inflation, increase statistical power, and allow the examination of potential interactions within a single statistical model.

##### Exploratory analyses

Consistent with the study protocol, all analyses beyond the primary EPN ANOVA were considered exploratory. These included tests on the effects of sex and models including covariates, (i.e., symptom change scores, pre-training trait anxiety (STAI X2) and childhood trauma (CTQ)), as well as interactions with diagnosis (MDD vs. HCS) in extended factorial models. Exploratory analyses were corrected for multiple comparisons. In the context of this, cut-off scores for depression severity subgroups were defined a priori as follows: BDI cutoffs (0–13: not depressed/in remission; 14–19: mild depression; 20–28: moderate depression; >29: severe depression;^52^). Subgroups based on HAMD-21 scores followed cutoffs: 0–7: no depression; 8–16: mild depression; 17–23: moderate depression, and ≥24: severe depression.^53^ Depression severity according to MADRS scores, followed original scoring instructions (0–6: no depression; 7–19: mild depression; 20–34: moderate depression; ≥35: severe depression.^43^

##### Data quality and sample

A total of 37 participants were excluded from EPN analysis due to insufficient EEG data quality or incomplete recordings. Exclusion rates were comparable across groups and treatment conditions: 19 HCS (9 PT, 10 CT) and 18 MDD (9 PT, 9 CT). EEG datasets were excluded when one or more of the following conditions were present: absence of valid event markers (e.g., S2 instead of S1, missing condition, missing response markers) incomplete paradigm execution (e.g., no EPN task performed, only resting-state EEG, missing second measurement), excessive signal contamination preventing meaningful ERP extraction (e.g., persistent noisy or unstable channels such as unresolved noise at F3, non-interpretable waveforms), insufficient valid behavioural responses or an insufficient number of analysable trials. Only datasets with sufficient event-related information and analysable signal quality were included, consistent with standard methodological requirements for ERP research.^54^ Baseline comparisons indicated no systematic demographic differences between excluded and included participants. Excluded HCS participants (mean age 39.47 years, 5 male/8 female) and excluded MDD participants (mean age 36.05 years, 8 male/12 female) were demographically similar to the final analysed sample. Because ERP-based neural outcomes cannot be validly estimated when no analysable event-locked EEG signal is available, an ITT approach was not feasible for EPN data. Imputation of EPN amplitudes is not possible without underlying neural data; therefore, the EPN dataset necessarily followed a quality-based inclusion approach. Importantly, exclusion rates were balanced across diagnostic and treatment groups, reducing the risk of systematic attrition bias.

##### Terminology

As the EPN, by definition, represents negative-going waves relative to neutral, negative, or less arousing stimulus conditions, a larger EPN refers to a higher negativity. In contrast, a reduced EPN refers to less negative mean amplitude data.

#### Clinical data

##### Clinical endpoint

The clinical endpoint was changes in MADRS scores (pre vs. post) in MDD. This secondary analysis was performed using linear mixed models, as they are robust to moderate non-normality, particularly in larger samples.^55^ Mixed Models were estimated using REML with Newton–Raphson optimisation. Fixed effects included diagnosis (MDD/HCS), training group (PT/CT), time (Baseline/Post/Follow-up), sex, and baseline BDI-II, HAMD-21, and MADRS scores. Depression scores were analysed to assess CBM training effects (PT/CT), including subgroup analyses by depression severity.

##### Further secondary clinical analyses

Pre–post and follow-up changes in HAMD-21 and BDI-II were also analysed using linear mixed models.

##### Moderator analyses

To explore potential moderators of symptom change, repeated-measures MANOVAs were performed with MADRS scores (Baseline, Post, Follow-up) as the within-subject factor and training condition (PT/CT) as the between-subject factor. Covariates included antidepressant use (MDD only), age, state anxiety (STAI-X2), and childhood trauma (CTQ). These moderator and subgroup analyses were exploratory. Non-parametric analyses (ArtTool)^56^; confirmed the results; only linear mixed model outcomes are reported in the main text for brevity.

##### Sample and missing data

The clinical dataset included 119 MDD (mean age 36.18 ± 1.178 years; 44 males/75 females) and 121 HCS (mean age 33.72 ± 1.18 years; 35 males/86 females). Missing data were handled according to an intention-to-treat (ITT) approach. Participants with insufficient EEG data quality were excluded from EEG analyses; baseline MADRS scores were comparable between included and excluded participants. For clinical outcomes and effects of sex, Mixed Model analyses were conducted. Change scores in MADRS, HAMD-21, and BDI-II (Baseline–Follow-up) served as dependent variables. EEG quality-based exclusion criteria and missing data handling procedures were implemented during data processing, given previous analysis experience (Fink et al., 2024).

### Role of funders

This study was funded by the “Deutsche Forschungsgemeinschaft” (DFG) (507720021) to authors Thomas Frodl and Bernhard Müller. The DFG had no role in the design of the study, the collection, analysis, or interpretation of data, or in the writing of this report.

## Results

Characteristics: Between February 2023 and October 2024, 390 participants were pre-screened; 242 entered pre-assessment (first patient in: 24.02.2023; last patient out: 19.12.2024). The trial was regularly concluded after the initial pre-assessment of the target sample size of 240. The final sample comprised 119 MDD and 121 HCS (PT: 62 MDD, 61 HCS; CT: 57 MDD, 60 HCS). Baseline demographic and clinical characteristics were comparable across groups. Tables 1 and 2 provides the baseline demographic and clinical characteristics for the four randomised groups. Age and sex distributions did not differ significantly across groups (age: *F*(3,236) = 1.333, *p* = 0.264; sex: χ^2^(3) = 5.480, *p* = 0.140). Clinical characteristics within HCS and MDD (BDI-II, HAMD-21, MADRS, STAI-X2) similarly showed no baseline differences between training conditions. Together, these findings indicate that the randomisation produced balanced groups at baseline. All randomised participants with baseline EEG data required for the calculation of the primary outcome (EPN) were included in ITT analyses. Missing data were imputed using last observation carried forward (LOCF). No serious safety concerns or unintended effects were reported. No adverse events required discontinuation or intervention. One expected adverse event occurred when a CT patient entered inpatient care from a pre-existing waiting list (see Table 5 for details on safety data). Overall, participant well-being remained stable throughout the study.

**Table 5:** Table of safety.

	Expected adverse events	Study emergent adverse events	Serious adverse events
Safety population (n = 240)	1	0	0
MDD PT (n = 62)	1	0	0
MDD CT (n = 57)	0	0	0
HCS PT (n = 61)	0	0	0
HCS CT (n = 60)	0	0	0

MDD = Major Depression Disorder; HCS = Healthy Control Subjects; PT = Positivity Training; CT = Control Training; No standard comparative effect sizes or 95% CIs are reported since only one expected adverse event occurred throughout the course of the study.

### Results of EPN analysis

The primary confirmatory analysis using independent-samples t-tests on post–pre EPN difference scores showed a statistically significant difference between PT and CT groups in MDD, t(97) = 2.24, *p* = 0.027, Cohen’s d = 0.45. The between-treatment difference in change scores was 1.65 μV (95% CI [0.19, 3.11]), indicating a greater shift towards more negative EPN amplitudes following positivity training. The mixed-design ANOVA in patients with MDD showed a statistically significant interaction effect between time (pre/post) and training (PT/CT) in patients with MDD, F(1,97) = 5.017, *p* = 0.027, partial η^2^ = 0.049, cohens *f* = 0.227. The between-treatment difference in change scores was 1.65 μV, 95% CI [0.19, 3.11]. EPN amplitudes shifted towards greater negativity in PT, while they shifted towards greater positivity in CT (see Fig. 2, Supplemental Table A4). In a secondary analysis, a statistically significant time∗training∗group interaction confirmed that this effect was specific to MDD, F(1,196) = 8.433, *p* = 0.004, η^2^ = 0.041. Post-intervention contrasts revealed larger treatment effects in MDD (mean difference = 0.88, 95% CI [−0.40, 20.17]) relative to HCS. No statistically significant time∗training interaction emerged in HCS. This finding was further supported by an exploratory time∗group interaction within CT participants, F(1,96) = 7.649, *p* = 0.007, η^2^ = 0.074, with larger EPN amplitudes in HCS and lower EPNs in the MDD group. Within CT, HCS showed decreased values from pre to post (estimated marginal means: 5.15 μV, 95% CI [4.03, 6.27] to 3.06 μV, 95% CI [2.21, 3.90], whereas MDD participants showed comparatively stable values (3.28 μV, 95% CI [2.14, 4.43] to 3.70 μV, 95% CI [2.84, 4.56]). In HCS, EPN amplitudes became more negative over time in both conditions, without interaction effects. None of the tested covariates (depression severity, changes in severity, medication status, STAI-X2, CTQ, study centre) revealed statistically significant effects. An effect of sex emerged in MDD (F(1,97) = 7.845, *p* = 0.006), with more negative EPN amplitudes in men than women. No sex differences were observed in HCS (Fig. 2C, Supplemental Table A5).

### Clinical outcome measures

**Fig. 3:**
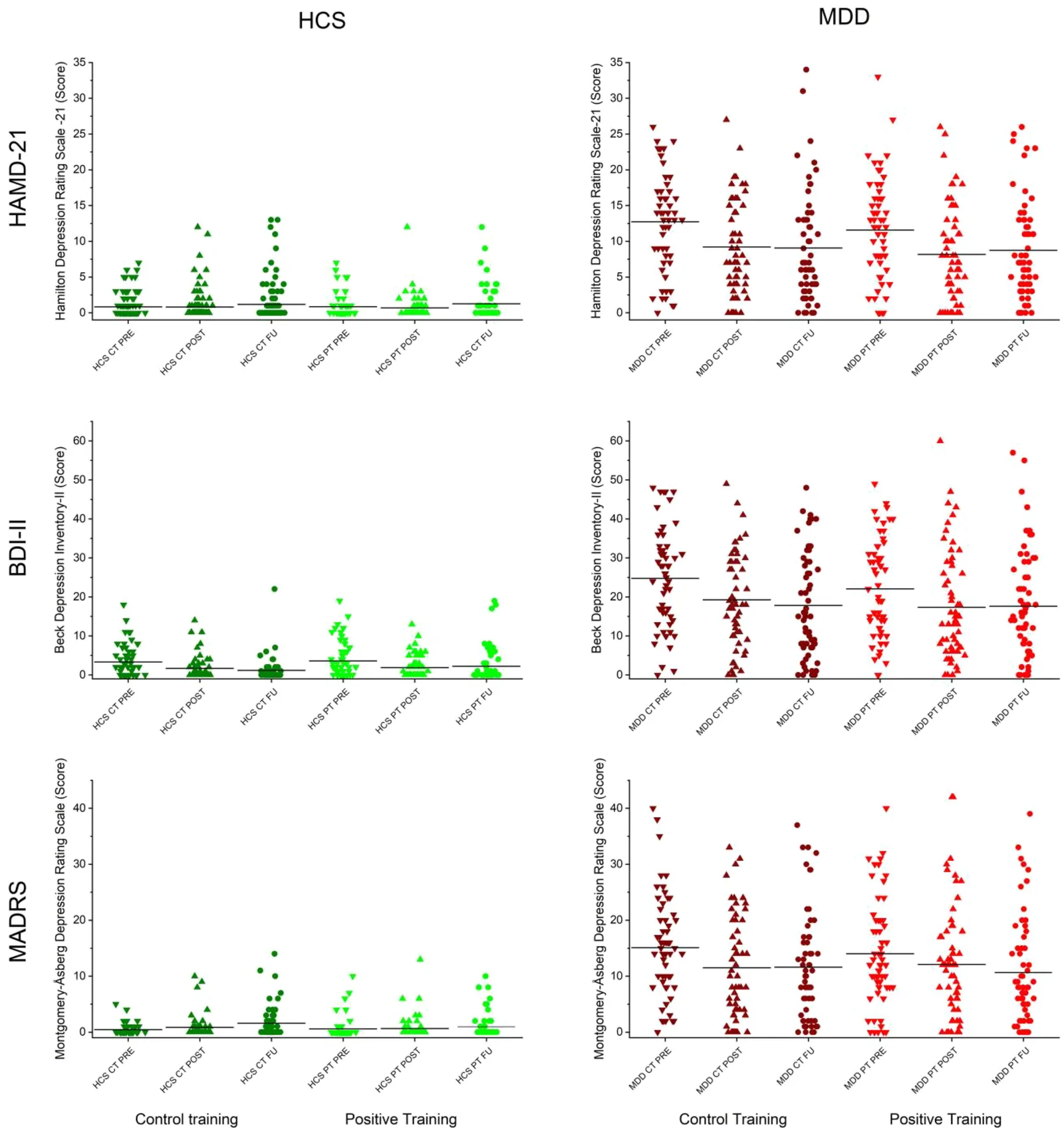
Changes in HAMD-21 (top), BDI-II (middle) and MADRS (bottom) scores in healthy control subjects (HCS) and Major Depressive Disorder (MDD) subjects from pre to post to follow-up. The panels illustrate individual data points for HCS and MDD subjects, respectively, across all time points and training conditions. Overall, MDD subjects showed reductions in depression severity from pre to post and from pre to follow-up across all three clinical measures, whereas HCS scores remained low and stable across time. Note. The x-axis represents time points (pre, post, follow-up), and the y-axis represents clinical test scores for each measure. HCS = healthy control subjects; MDD = Major Depressive Disorder subjects; CT = control training; PT = positivity training; BDI-II = Beck Depression Inventory–II; HAMD-21 = 21-item Hamilton Depression Rating Scale; MADRS = Montgomery–Åsberg Depression Rating Scale. Individual panels additionally display raw data distributions. Group differences between training conditions (CT vs. PT) were examined separately within each group (HCS, MDD).

Mixed-model analysis unveiled a statistically significant interaction between group and time F(2/197) = 10.10, *p* < 0.001, indicating a reduction in depressive symptoms according to MADRS over time in patients with MDD. Compared to HCS, patients with MDD showed higher MADRS scores at all time points, with mean differences of 13.69 (95% CI [11.77, 15.62]) at baseline, 10.81 (95% CI [8.88, 12.74]) at post-intervention and 10.32 (95% CI [8.33, 12.31]) at follow-up (Fig. 3).

#### Secondary clinical symptom analyses

Mixed Model analyses indicated interactions between group and time for HAMD-21 (F(2/197) = 15.16, *p* < 0.001), compared to HCS. Patients showed significantly higher HAMD-21 scores at all measurement occasions, with mean differences of 11.05 (95% CI [9.64, 12.47]) at baseline, 8.24 points (95% CI [6.63, 9.85]) at the second assessment, and 7.62 (95% CI [6.06, 9.19]) at follow-up and BDI-II (F(2/197) = 10.10, *p* < 0.001), with mean differences of 19.24 (95% CI [16.61, 21.88]) at baseline, 15.55 points (95% CI [12.89, 18.20]) at the second assessment, and 14.72 points (95% CI [11.90, 17.54]) at follow-up. In both cases, the statistically significant interactions indicated significant reductions in depressive symptoms in patients over time. See Appendix B, Supplemental Table A6 and A7 for non-significant findings.

## Discussion

This clinical trial demonstrated that active CBM positivity training was significantly associated with patients’ neurophysiological responses to positive emotion stimuli. A significant interaction between time and training group was observed. Patients with MDD demonstrated a larger EPN after PT, and a reduced EPN after CT. An increased EPN with more negative amplitudes implies increased neural activity in response to visually presented positive stimuli in patients with MDD.^57^ This suggests PT was associated with changes in emotional processing by enhancing neural sensitivity to positive information, aligning with the training’s aim. In this study, we investigated CBM training effectiveness utilising EPN as a neurophysiological marker in a large cohort of MDD and HCS. We found that EPN changes were specific to patients in PT. Patients might rely on a consistent attentional re-direction to positive material, as in CT, containing only a 50% shift towards positive stimuli, no EPN changes were observed. In contrast, HCS reacted to even a partial attentional re-direction, evident in their shift towards negativity in EPN pre- to post-training, regardless of training group. As more studies will be needed, these findings should also be interpreted within a hypothesis-generating framework regarding the mechanisms behind CBM.

Medication intake did not affect EPN outcomes, suggesting that the positivity training’s effects in patients occurred independently of medication. Furthermore, women with MDD showed more negative EPN than men, while training effects were consistent across sex in patients, underscoring the usability of active CBM training.

As an ERP component, EPN reflects early, automatic attentional engagement with emotionally salient stimuli.^23^, ^24^, ^25^, ^26^ ERPs provide high temporal resolution and index early, automatic emotional processing stages not captured by behavioural or self-report measures. The EPN is sensitive to subtle, training-induced modulations in attentional engagement with emotional stimuli, potentially detecting neural changes before observable clinical improvements. Recent work highlights the translational utility of ERPs as biomarkers for monitoring mental health treatment effects, supporting their role as objective indicators of clinical change.^58^

The underlying neuronal mechanisms of changes in EPN remain underexplored. Lower glutamate levels in the prefrontal cortex of patients with MDD have been reported.^59^ A recent experimental glutamate challenge in healthy women revealed short-term effects on positive emotions.^60^ Although glutamate neurotransmission has been associated with frontal P3 ERP,^61^ its relationship with EPN remains to be investigated. Given the limited research on EPN and the scarcity of reports on potential interactions between EPN and other emotion processing indicators, future studies must investigate the biological basis underlying the treatment-specific EPN effect in patients and the general effect in controls.

The observed shift towards negativity in EPN implies that PT was associated with altered neural responses to emotionally positive content. However, no significant benefit of PT over CT was found in terms of improvement in depression severity, suggesting that these neurophysiological effects may be more function specific and occur before or beyond clinical improvements. This implies that neurophysiological markers, such as EPN, could serve as early surrogate markers of changes following active CBM training. The National Institute of Mental Health’s RDoc framework supports integrating multiple data types (e. g., self-ratings, neurophysiology, etc.) for a comprehensive understanding of psychopathology and healthy functioning.

Interestingly, symptoms of patients with MDD significantly declined over time, independent of training condition, supporting prior results of Vrijsen et al.^18^ Thus, engagement in the training itself could promote a general positive cognitive shift, as both training conditions promoted a shift towards positive stimuli, differing only in intensity (PT: 100%, CT: 50%). Additionally, participation itself might have positively influenced symptom reduction via increased social interaction with psychologists and structured routines, promoting indirect therapeutic and motivational effects.^62^ This idea is supported by meta-analyses revealing placebo effect sizes for primary outcomes ranging from 0.23 to 1.85 in psychiatric interventions.^63^

Overall, the results concerning the reduction of depressive symptoms are consistent with mixed findings in previous studies, with some observing clinical improvements following CBM training, while others detected no effects.^13^^,^^14^^,^^16^^,^^64^ The variability in training tasks and frequencies may explain heterogeneous results. Thus, the overall lack of a significant superiority of PT vs. CT in the present study might also trace back to the training setup. Follow-up studies comparing conditions with different training frequencies, intensities, and lengths will provide valuable insight into necessary training characteristics for translating reported EPN changes into clinical improvements. Sex, age, and state anxiety did not significantly influence symptom changes, and only small effect sizes were observed concerning medication status, suggesting minimal effects, possibly due to sample size.

The general decline in depressive symptoms across training groups may partly reflect regression to the mean or non-specific factors such as engagement in structured tasks and social interaction. Importantly, the observed EPN changes in patients with MDD were training-specific and do not merely reflect overall symptom changes. Prior studies, such as Xin et al.^29^ reported reduced EPN amplitudes in depressed participants at baseline without a shift towards positive stimuli, whereas our results demonstrate training-induced modulation of EPN. Differences in sample characteristics, task design, and analytic approaches may account for these discrepancies. Future studies employing multimodal imaging approaches could help clarify the neural mechanisms linking EPN modulation to depression pathophysiology.

The positive findings concerning EPN changes only in patients with MDD after CBM mark a notable result in the current state of EPN and CBM research. EPN seems to be a gateway for investigating positive-valence information processing and its change over time, echoing previous findings in psychooncology.^30^ The study reported no serious adverse events and was conducted with limited patient contact. A limitation is that it was confined to two centres, primarily involving patients with mild to moderate depression, without a comparative arm alongside the non-active control training. The absence of structured adverse event monitoring restricts formal safety analyses. Future confirmatory studies should predefine benefit and harm outcomes and apply standardised safety documentation. Moreover, future studies should add a third EEG measurement during follow-up (e.g., 6–8 weeks post-intervention) to explore the persistence of EPN amplitude changes and their association with sustained clinical improvements. The reported results cannot be confidently generalised across different ethnic groups, since ethnicity was not specifically assessed at inclusion. Moreover, participants may have partially inferred their assigned training condition due to differing arrow contingencies between PT and CT, suggesting the need for formal assessments of blinding integrity. The present study demonstrates the successful implementation of ERP-based assessments within a randomised clinical trial and provides important methodological insights for optimising future trial design, including measurement timing, sample size planning, and intervention paradigms. We propose that further studies be conducted, potentially including a no-training or neutral/negative stimuli condition. In contrast, patients with severe depression may require tailored intervention programmes, regarding the frequency and content. Ideally, future studies should also add EPN as a neurophysiological measure, as it may serve as a surrogate marker for neural changes in depression, which may facilitate its utilisation in both industrial and clinical studies to assess the efficacy of interventions. Further studies are needed to clarify the clinical relevance of EPN as a neurophysiological marker. Effect sizes from this study should be interpreted cautiously as initial effect size estimates are often subject to upward bias. Accordingly, the effect sizes reported here should be interpreted with caution when considering future study designs.

For conclusion, CBM positivity training was associated with treatment-specific neural effects in MDD. Further studies with another follow-up EEG, higher stimulus valence contrasts in the training conditions, and systematic benefit and harm assessments are needed to clarify clinical relevance and safety.

## Contributors

Zoe Kratochwil & Jessica Kesik: Data curation, project administration, resources, investigation, visualisation, formal analysis, interpretation of results, writing-original draft. Both authors hold Master’s degrees in Psychology, are pursuing PhDs, and conducted the SPSS analyses under supervision. Thomas Frodl & Bernhard Müller: Conceptualisation and design. The data presented in this paper have been primarily accessed, verified and analysed by Zoe Kratochwil, Jessica Kesik, Thomas Frodl, and Bernhard Müller also accessed and verified the data to verify proper data handling. Funding acquisition, resources, supervision, data analysis, interpretation of results, writing & editing, validation, methodology. Dr. Frodl is a psychiatrist and experienced clinical researcher, and Dr. Müller a psychologist with expertise in medical statistics and experimental methodology. Both supervised and reviewed the statistical analyses. Duygu Keskin-Gökcelli: Participant investigation including clinical data and EEG, interpretation and discussion of results, editing and reviewing of the manuscript. Norbert Scherbaum: supervision of clinical trial, editing manuscript. Katrin Sakreida: software (EEG resting state, Dot-probe task). All authors read and approved the final version of the manuscript.

## Data sharing statement

Study data will be utilised for the present project in the first place and data will be stored on local computers including local data backups. Data will be analysed and published primarily by PhDs associated with the study and the PI’s. For researchers, de-identified data are available upon reasonable request to the corresponding author (tfrodl@ukaachen.de), subject to the approval of a brief research proposal and a signed Data Use Agreement to ensure participant privacy. In addition, study data will be submitted to a repository (DUE publico, https://doi.org/10.71955/DUEDATA-2026-MRT6IZNE) to store study data including EEG-data and SPSS data to allow further use of the study data in anonymised form after research questions have been analysed and published. The above-mentioned contact can be used.

## Declaration of interests

The authors report no competing interest with respect to the study and the content of the manuscript. Prof. Frodl is president of the German medical Society for Behavioural Therapy (DÄVT) and of the Society to Promote empirical based therapy in schizophrenia (gfts). Dr. Sakreida has received compensation for providing a training course on transcranial magnetic stimulation for the treatment of depression.

## Appendix A Supplementary data

Supplemental Tables

Projektplan_22_065
