# Supplementary material for: A tablet-based attentional cognitive bias modification intervention for positive emotion processing in major depressive disorder: a single-blind randomised controlled two-centre trial
Source: eBioMedicine. 2026 Sep 1;132:106453. doi: 10.1016/j.ebiom.2026.106453 (PMC13570274; doi:10.1016/j.ebiom.2026.106453)

Supplemental Figure 1

CBM app implementing the dot-probe task. In PT, arrows followed positive images; in CT, arrow positions were random (50 % neutral or positive). Each trial began with a 1000 ms fixation cross, then one neutral and one positive image were presented for 500 ms (one above the other). After the image offset, an arrow (left/right) appeared at the place of one of the two images, and participants tapped the indicated screen side. Feedback (green check/red cross) was shown for 500 ms. Each session included eight practice and 240 training trials, organized in blocks of 40. Pre−training trials to assess positivity bias were included in sessions one, six, and ten. Participants and clinical raters were blinded to training condition (PT/CT).

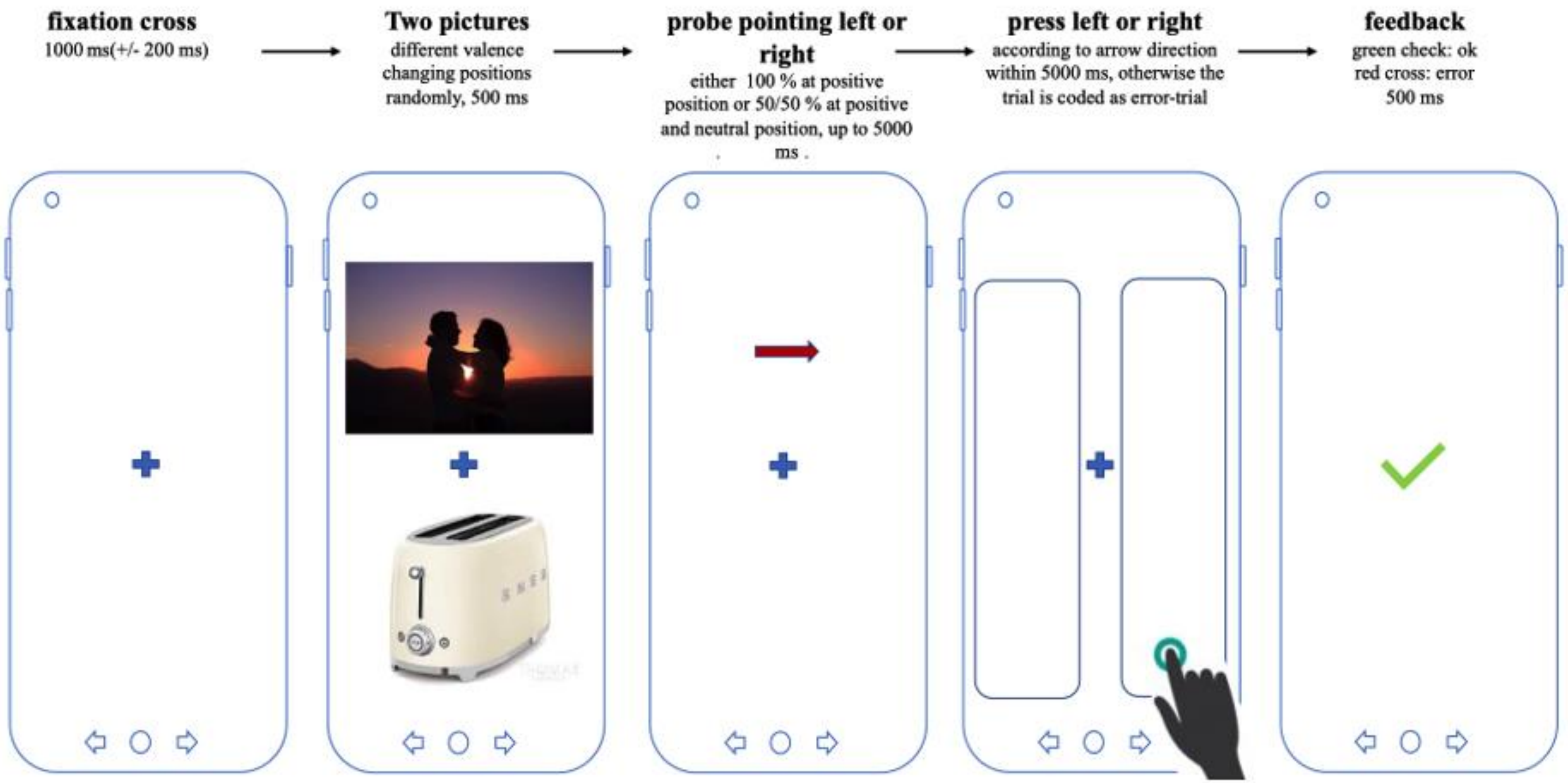

Supplement: Supplementary file 1 [file mmc1.pdf]
