# Supplementary material for: A tablet-based attentional cognitive bias modification intervention for positive emotion processing in major depressive disorder: a single-blind randomised controlled two-centre trial
Source: eBioMedicine. 2026 Sep 1;132:106453. doi: 10.1016/j.ebiom.2026.106453 (PMC13570274; doi:10.1016/j.ebiom.2026.106453)
Supplement: Supplementary file 2 [file mmc3.pdf]

1. Projekttitel, Versionsnummer, Versionsdatum

Positivity Training: Effects of a Bias Modification Training on Emotion Processing in Major Depression and healthy Controls

Positivitäts-Training: Effekte eines Kognitiven-Bias Trainings auf Emotionsverarbeitung bei Major Depression und gesunden Kontrollen

Version 1.0 vom 11.02.2022

2. Zusammenfassung des Projekts

Bei Menschen mit Depression beeinflussen negative Schemata die Informationsverarbeitung nicht nur indem sie die Aufmerksamkeit auf negative Ereignisse richten, sondern auch in dem sie die Verarbeitung positiver Ereignisse verändern. In einer vorauslaufenden Studie konnten wir unter Verwendung einer Dot-Probe Aufgabe zeigen, dass ein Training zur impliziten Fokussierung auf positive Ereignisse, depressive Symptome verminderte.

Die aus dem EEG abgeleitete Early Posterior Negativity (EPN) hat sich in neueren Untersuchungen als sensitiv für frühe, automatisierte Emotionsverarbeitung gezeigt. Ganz aktuell konnte dargestellt werden, dass Patienten mit Major Depression gegenüber Kontrollpersonen eine verminderte EPN für Gesichter mit positiven Emotionen zeigen. In einer noch laufenden Studie, können wir die spezifische Sensitivität der EPN für die Verarbeitung emotional positiver Stimuli mit eigenen Daten bestätigen.

In dieser experimentellen, randomisierten, einfach verblindeten Studie möchten wir daher bei 120 ambulanten Patienten mit depressiver Störung und bei 120 gesunden Probanden ein App-basiertes Positivitätstraining im Vergleich zu einem Kontrolltraining durchführen und diese experimentelle Intervention als Ausgangspunkt für die Untersuchung von veränderter Informationsverarbeitung auf positive Stimuli verwenden.

Die Teilnehmer werden über 2 Wochen in zehn Einheiten selbstständig ein App-basiertes Positivitätstraining auf der Basis einer Dot-Probe Task durchführen, das je Einheit 25 Minuten dauert. Klinische, und EEG-Maße werden vorher und nachher untersucht. Ein klinisches Follow-up ergänzt die Einschätzung der Interventionseffekte. Bei allen Patienten wird die sonst durchgeführte ambulante, leitlinienkonforme Standardbehandlung weitergeführt. Das primäre Zielkriterium ist dabei die Veränderung der EPN in der Verarbeitung positiver Stimuli.

Wir erwarten, dass durch das Positivitäts-Training insbesondere automatisierte Verarbeitungsprozesse von positiven Stimuli bei Depression verändert werden. Die Untersuchung weiterer EEG-Komponenten der Informationsverarbeitung und von EEG Microstates wird es erlauben die Effekte automatisierter, früher Informationsverarbeitung von mehr aufmerksamkeits-abhängigen Prozessen abzugrenzen und damit die biologische Basis solch eines Positivitäts-Trainings bei Depression im direkten Vergleich zu gesunden Kontrollprobanden besser zu verstehen.. Für die Beschreibung der Hintergründe und die Details zur Studiendurchführung wird auf den beigefügten Forschungsantrag Bezug genommen.

3. Verantwortlichkeiten

⍰ Studienleiter/in

Prof. Dr. med. Thomas Frodl
Universitätsklinik für Psychiatrie und Psychotherapie, Medizinische Fakultät
Universitätsklinikum Aachen
Direktor der Klinik
0241 80-89633
tfrodl@ukaachen.de

⍰ beteiligte Wissenschaftler/innen

PD Dr. phil. Bernhard Müller
Klinik für Psychiatrie und Psychotherapie,
LVR-Klinikum Essen, Klinik der Universität Duisburg-Essen
Ltd. Psychologe
0201 7227 250
bernhard.mueller@uni-due.de

▯ beteiligte Einrichtungen

n.A.

▯ Finanzierung

Deutsche Forschungsgemeinschaft

▯ Registrierung in einem öffentlich zugänglichen Studienregister

DRKS / WHO

4. Wissenschaftlicher Hintergrund

▯ Stand der Forschung (mit Literaturangaben) und Ableitung der Fragestellung (Rationale)

Übernommen aus DFG-Antrag (in Englisch):

1.1 State of the art and preliminary work

Major Depressive Disorder (MDD) is the most common psychiatric disorder and is associated with a tremendous burden of disease not only for the patients but also for those who care for them. Depression is the leading cause in the EU of lost work productivity, disability, long-term sick leave, and early retirement. Indeed, according to the WHO, depression is now the leading cause of disability worldwide. In addition to the human burden, depression is the costliest brain disorder in Europe, accounting for 1% (€118 billion annually) of the total European economy. Depression can be very difficult to treat. Medication and cognitive therapy are effective, however about 30% of patients (i.e. 9 million patients in Europe alone) do not respond to standard treatments even after multiple treatment steps.

Cognitive theories of depression have convincingly conceptualized schemas on negative beliefs of the self, the personal world and the future (Beck, 1967). The most prominent cognitive model has been introduced by Beck and has frequently been updated to include accumulated evidence (Beck, 2008; Beck & Haigh, 2014). Beck's cognitive model of depression builds upon the pronounced clinical consequences of altered physiological, attention, and memory functioning related to preferential processing of negative information. Negative schematas influence information processing by orienting salience to negative events and decreasing attention to positive events (I. H. Gotlib, Neubauer, D.L. , 2000)

However, next to these so-called negative biases, changes in positive affect and dysfunctional processing of positive information in itself may form an active process leading to negative affect and supporting negative biases. Indeed, in their tripartite model of depression and anxiety, Clark and Watson formulated, among others, low positive affect as an important dimension characterizing depression and distinguishing anxiety from depression (Clark & Watson, 1991). This positive dimension has been updated to a positive valence system in the context of the RDoC-like (Research Domain Criteria, National Institute of Mental Health) dimensional approach (Watson, 2009).

Depression und negative memory bias

Negative bias in depression is well known and goes back to an early report by Gotlib and McCann (I. H. Gotlib & McCann, 1984), who were the first to show prolonged response latencies with negative mood words as compared to neutral words and in comparison to a non-depressed control group. The relevance of biased processing in depression has since then been extensively studied as summarized in various reviews (I. H. Gotlib & Joormann, 2010; Miskowiak & Carvalho, 2014) and meta-analyses (Peckham, McHugh, & Otto, 2010). Biased processing in depression has been found for various cognitive domains, ranging from attention to interpretation and memory (Rinck & Becker, 2005). Generally, for group comparison between patients with MDD and healthy controls a medium effect size of 0.52 for studies assessing responses to negative faces or words and smaller effect sizes for studies using dot-probe or emotional stroop tasks have been found (Peckham et al., 2010).

Of the various cognitive domains, in which a negative bias can be tested, negative memory bias is the most relevant cognitive risk factor for the development, maintenance, and recurrence of depression (De Raedt & Koster, 2010; I. H. Gotlib & Joormann, 2010). Notably, Vrijsen and colleagues have shown the link between memory bias and genetic susceptibility for depression (Vrijsen et al., 2015).

Depression and biased processing of positive stimuli

With regard to the processing of positive stimuli, experimental results have been attributed to a form of "evenhanded" shift in the processing of emotion related stimuli in subjects with depression. Changed processing of positive information in depression is not only **a shift towards more mood congruent negative processing, but can be formalized as an active process of inhibition of rewarding stimuli by devaluation of these positive stimuli** (De Vito, Al-Aidroos, & Fenske, 2017; Frischen, Ferrey, Burt, Pistchik, & Fenske, 2012; Winer & Salem, 2016). Taken together these studies point towards a specific role in the processing of positive information in patients with MDD. Indeed, altered neurocognitive positivity processing may even last when being in remission from depression (see for example altered positive memory bias in Arnold et al. (2011)).

ERP to demonstrate negative processing bias

Event-related potentials (ERP) are a type of long-latency evoked potentials extracted from ongoing electrical cerebral background activity by averaging related procedures to reflect human information processing. ERP has a high time resolution, which is important when investigating automated information processing with regards to emotion bias. There are two main ERP markers for emotional attention, the early posterior negativity (EPN) and the late positive potential (LPP).

Processing of high-arousing pleasant or unpleasant compared with low-arousing control images is associated with a negative-going potential over temporo-occipital sensor regions around 150–300 ms poststimulus and this is called the EPN. This occipital negativity effect may reflect a transitory processing period within which emotional stimuli are selected for elaborate processing (Junghofer, Bradley, Elbert, & Lang, 2001; Schupp, Junghofer, Weike, & Hamm, 2003). It has been shown that the EPN is greater for positive than for negative stimuli (Schupp et al. 2004). In near infrared spectroscopy significant differences of oxygenated hemoglobin indicating increased cerebral blood flow, e.g. due to regional brain activation, was found between the positive and neutral condition and not between the negative and neutral condition. However, these changes were not directly correlated with EPN amplitudes (Herrmann et al., 2008).

Studies investigating EPN are limited in the research field of MDD. A very recent study by Xin, Yu, and Zhao (2021) reported that in controls, the EPN was significantly enhanced for positive as compared to negative emotion faces, and that this emotional effect was not evident in MDD patients. There are some additional reports from related fields. The amplitude of EPN was significantly less negative in participants with burnout syndrome (N=47) compared to those without burnout (N=46) (Golonka, Mojsa-Kaja, Popiel, Marek, & Gawlowska, 2017), whereby no interactive effect between group and stimulus type (negative, positive, neutral) occurred. This change of EPN amplitude during burnout is in

the same direction as the influence from emotional stimuli, so that we hypothesize that in depression we also might see less negative EPN amplitudes. Larger EPN differences between high-arousing positive and low-arousing neutral IAPS pictures were also found in adolescents exposed to various amounts of traumatic stress (Sill, Popov, Schauer, & Elbert, 2020).

The P300 component of the ERP occurs at 300 ms to 600 ms post stimulus as a positive deflection, which is largest at parietal electrodes when stimuli are task-related. It was first described by Sutton, Braren, Zubin, and John (1965) and frequently studied the following decades (Polich & Kok, 1995). More recently, the LPP has been assessed in the latency range of the P300 component (Cuthbert, Schupp, Bradley, Birbaumer, & Lang, 2000). In contrast to the P300 component, the LPP has been more closely related to emotion processing (Hajcak, MacNamara, & Olvet, 2010) and is found when arousing (positive or negative) stimuli are presented compared to neutral stimuli (Schupp, Schmalzle, & Flaisch, 2014). Methodically the LPP is determined as a mean amplitude within a given latency interval. Depending of the task, this may be at between 400 to 600 ms post stimulus onset, but recent studies discriminate between an early (400 – 600 ms), a middle (600 – 800 ms) and a late (800-1000 ms) time window (Schindler & Straube, 2020; Weinberg, Hilgard, Bartholow, & Hajcak, 2012). While the theoretical differentiation between P300 and LPP is still under discussion, in a recent review Hajcak concludes, that both reflect "output from a common system that tracks the time-course of stimulus significance" (Hajcak & Foti, 2020).

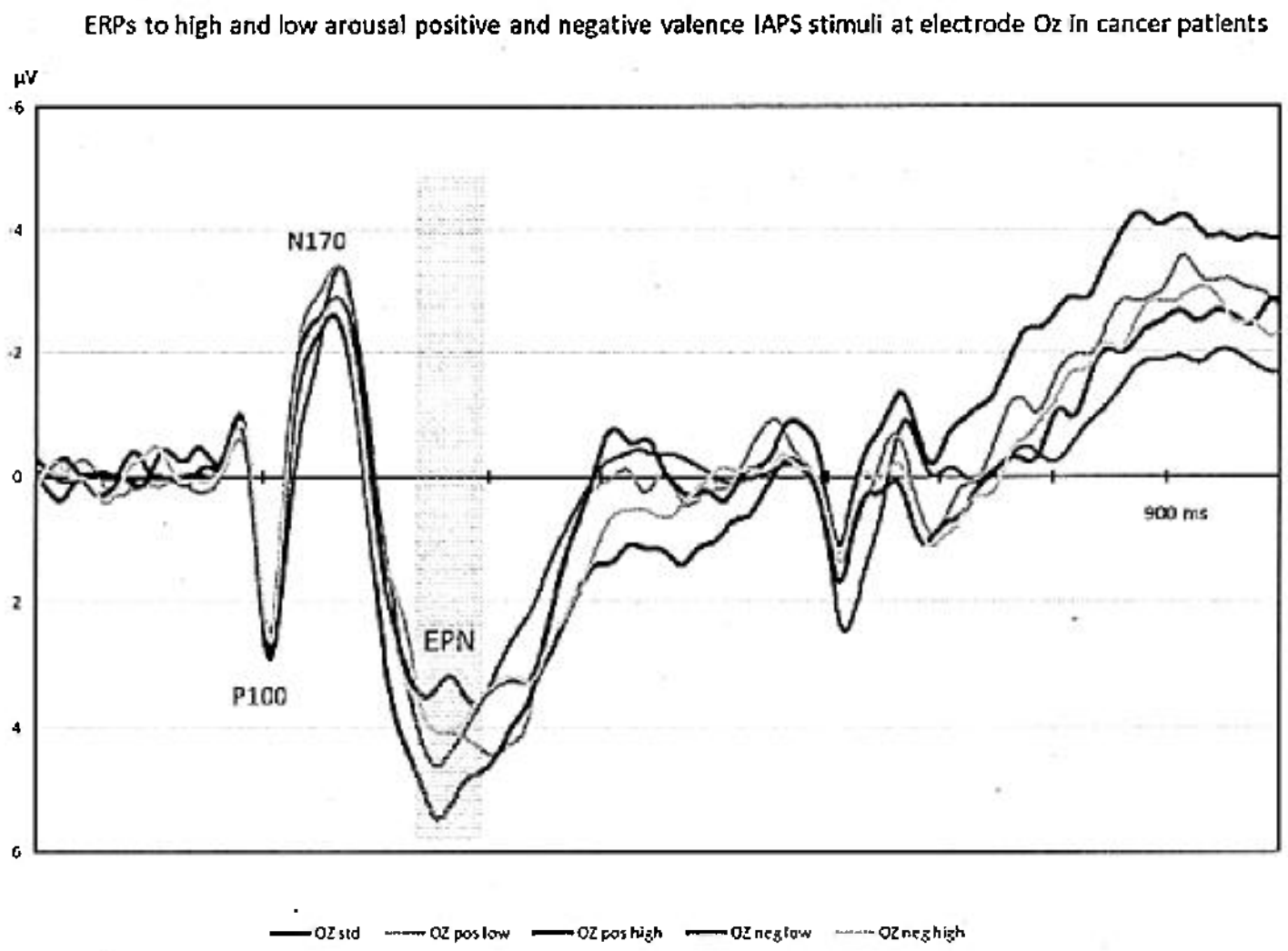

Figure 1

Regarding stimulus valence in LPP, positive stimuli may result in larger positive deflections at frontal and central electrode positions when adjusted for the arousal dimension (Cuthbert et al., 2000). In MDD, lower LPPs to emotion stimuli have been reported when compared to controls. Of specific relevance to our experimental intervention, the LPP has been found to be reduced to pleasant stimuli and smaller LPP amplitudes were associated with increased anhedonia severity in MDD. (Klawohn, Burani, Bruchnak, Santopetro, & Hajcak, 2020). (Bondy et al., 2018) found the LPP in children to positive and negative stimuli to be stable over time and to show good internal consistency. In adolescent girls, a low LPP was associated with increases in depressive symptoms when subjects experienced live stressors. This moderating effect of LPPs to positive valence stimuli was larger than those to negative (Levinson, Speed, & Hajcak, 2019). Additionally, preschool children with depression symptoms showed reduced LPP to positive as compared to neutral stimuli (Whalen et al., 2020). In adult patients with MDD Hill and colleagues found reduced LPP to negative as well as positive-valence stimuli when using structural equation modeling on ERP data (Hill, South, Egan, & Foti, 2019). Regarding MDD treatment, Nandrino (2004) detected that P300 amplitudes to positive words in first episode patients remained decreased after clinical improvement. In another study in children, LPP amplitude to pleasant stimuli remained low, while larger pre-treatment LPPs to positive stimuli were associated with remission from depression (Barch et al., 2019).

Currently, in cooperation with Prof. Teufel (Clinic for Psychosomatic Medicine at the University of Duisburg-Essen), we investigate emotion processing in cancer patients. In an oddball task we use neutral, high and low arousal positive and negative valence stimuli from the IAPS series and are able to access P100, N170, LPP and EPN. In the high arousal conditions, the EPN demonstrates differential

ERPs to positive and negative stimuli (Figure 1). The high arousal positive valence stimuli lead to more negative amplitudes in the range of 220 ms to 280 ms, which is in the latency range of the EPN. Figure 1 shows grand average ERPs from 41 assessments in these patients in reaction to neutral and high and low positive and negative valence stimuli presented for 500 ms with 2500 ms intertrial interval. The EPN can be computed as difference of emotional stimuli minus the neutral standards and therefore the EPN after positive high arousal stimuli is larger (i.e. more negative) than the EPN to neutral or low arousal negative stimuli.

While the group around Hajcak has gathered quite some evidence on the relevance of the LPP in MDD, from a theoretical perspective the EPN is of special interest to our application. The EPN indexes early differential processing of emotional as compared to neutral visual stimuli (Schupp, Flaisch, Stockburger, & Junghofer, 2006). It is theoretically closer than the LPP to more automatic emotion processing which is targeted by the Cognitive Bias Modification (CBM) positivity training to be further evaluated here (Vrijsen et al., 2018). Furthermore, our own data indicate a specific sensitivity to the processing of positive valence stimuli. Therefore, here we will focus on the EPN to positive stimuli as primary ERP outcome parameter.

Resting State EEG

There is evidence that increased frontal alpha asymmetry is a reproducible candidate marker for MDD diagnosis (Thibodeau, Jorgenson, Kim 2016). Frontal asymmetry has been related to the processing of positive and negative emotions in both trait and state emotion processing (Coan & Allen, 2004). Frontal alpha activity seems to be commonly increased in MDD and to a lesser extent also beta 3, delta, theta, beta 1 and beta 2 activity (Korb, Cook, Hunter, & Leuchter, 2008). On the other hand, decreased alpha 1 and beta 2 activity was found in parietal lobes in patients with MDD compared to controls (Pizzagalli et al., 2002). Moreover, increased gamma band activity was demonstrated at rest and during task activation in MDD (Siegle et al. 2010, Strelets et al. 2002).

During an EEG recording, a specific topography (or "microstate") persists for tens of microseconds, and then transitions to a different topography. Advancements in clustering algorithms and computing power have allowed for more precise examination of microstates (Lehmann & Michel, 2011). Microstates are generated by distinct, but overlapping, neural assemblies and their parameters and topographical transitions provide information about large-scale brain connectivity (Milz, Pascual-Marqui, Achermann, Kochi, & Faber, 2017). There is still a matter of debate whether microstates correspond to functional MRI resting state networks, or whether these represent distinct information. There is limited research capitalizing on microstate analysis to investigate affective disorders, and in particular on MDD. Duration and occurrence of microstate D was reduced in MDD compared to healthy controls. Among patients with MDD duration and occurrence of microstate D were negatively correlated with symptom severity. Furthermore, patients with current MDD exhibited multiple distinct transition probabilities compared to healthy controls and patients in remission (Murphy et al., 2020). Interestingly, microstate D might be associated with the fronto-parietal/attention network. In this way, it might be associated with attention-related evoked potentials like EPN and LPP.

To our knowledge no study investigated the effect of cognitive bias modification on EEG microstates, although these are related to large-scale brain networks.

Bias modification in depression

Over the last decade several experimental techniques have been developed to directly alter maladaptive cognitive biases in order to restore distorted information-processing (Macleod & Holmes, 2012). This method is commonly referred to as Cognitive Bias Modification (CBM). Among the most promising CBM techniques for depression are paradigms that aim at modifying the tendency to interpret ambiguous stimuli or scenarios in a benign rather than a negative way (for review see Menne-Lothmann et al., 2014)). Besides other forms of CBM, using generally positive stimuli can increase approach of positive materials and decrease emotional reactivity in dysphoric individuals (Becker et al., 2016) as well as symptoms in depressed patients (Vrijsen et al., 2018).

Recently, we extended these findings to a clinical sample of depressed patients and investigated the effects of a positivity increasing attention training and a positivity-approach training compared to a control training in a blinded randomized controlled design. 139 depressed inpatients received either four sessions of a CBM Attention Dot-Probe Training (DPT) or a CBM Approach-Avoidance Training (AAT), next to treatment as usual. Clinician-rated depressive symptom severity decreased significantly more in patients, who received the active condition of the DPT or the AAT, compared to patients in the control condition (Vrijsen et al., 2018). In this study, the dot-probe task was associated with a significant improvement in the positivity-bias, which, however, was not specifically related to symptom improvement. More recently, the relevance of a training for positive stimuli was confirmed at follow-up in MDD patients using the dot-probe task as an add-on treatment with 10 daily training sessions (Dai, Hu, & Feng, 2019). Therefore, here we will focus on the dot-probe task in order to study its effects on emotion processing using EEG/ERP markers.

5. Projektziele

▯ primäre/sekundäre Ziele

Primäres Projektziel ist die experimentelle Untersuchung der EPN Komponente des visuell evozierten Potentials im Kontext eines Positivitätstrainings bei Patienten mit MDD und bei Kontrollen. Wir erwarten, dass die EPN als Indikator eher automatisiert ablaufenden Emotionsverarbeitung in der Gruppe der Patienten mit aktivem Positivitätstraining verbessert, d.h. in der Amplitude (ihrer Negativität) erhöht wird.

6. Zielgrößen

▯ primäre/sekundäre Zielgrößen

Primäres Zielkriterium der Studie ist die Veränderung der aus dem EEG abgeleiteten Early Posterior Negativity (EPN) Amplitude in der Verarbeitung positiver Stimuli mit höherem Arousal vor- und nach dem Positivitäts-Training. Weitere Zielgrößen sind die Veränderung des so genannten Late Positive Potential (LPP) und von so genannten EEG-Microstates, die aus dem Resting-State EEG abgeleitet werden. Weiteres sekundäres Zielkriterium ist die Verbesserung der Depressionssymptomatik (MADRS-Scores) in der Gruppe mit dem Positivitätstraining, gegenüber der Gruppe mit dem Kontrolltraining und gegenüber gesunden Kontrollprobanden. Zusätzliche sekundäre Analysen betreffen die Bedeutung von bisherigen Lebenserfahrungen, aktueller Symptomatik und kognitiven Leistungen für die ERP-Maße der Emotionsverarbeitung, das LPP und die EEG-Microstates.

The description of the work programme applies to both study centers: The project is planned as cooperation between the sites at Aachen and Essen in Germany, since this will allow for a faster recruitment and larger sample. The work programme regarding participant recruitment, assessments and intervention will be the same at both sites. Data analysis will be done cooperatively but with focus on LPP and EEG microstate analysis in Aachen and EPN data analysis in Essen. The primary clinical outcome parameter (change in MADRS sores) will be assessed by experienced raters blind to the intervention group.

7. Studienpopulation

▯ Ein- und Ausschlusskriterien

- Einschluss alle: Alter: 18-60 Jahre
- Einschluss Patienten: Major Depression (DSM-V 32.0/1/2/4/5/8/9; 33.0/1/2/4/5/9) in ambulanter Versorgung

- Ausschluss alle: Aktuelle psychotische Symptome, Bipolare Störung, Akohol- oder Substanzabhängigkeit (außer Nikotin). Neurologische Erkrankung mit Einfluss auf Hirnfumktion (z.B. Schilddrüsenerkrankungen, dysregulierter Diabetes (HBA1C > 7.5) , Schlaganfall). Schwangerschaft

□ ***Anzahl der Studienteilnehmer***

120 Patienten: 60 Aachen + 60 Essen, 60 Kontrolltraining, 60 experimentelles Training

120 Kontrollen: 60 Aachen + 60 Essen, 60 Kontrolltraining, 60 experimentelles Training

□ **Rekrutierungsmaßnahmen (wo und wie werden die Teilnehmer rekrutiert?)**

- Mögliche Studienpatienten werden über Aushänge im Umfeld der Klinik rekrutiert. Die Kontrollprobanden werden über Aushänge rekrutiert.
- Informationsschriften, Einwilligungserklärungen und Rekrutierungsmaterialien liegen als Anhang bei.

8. Methodik und Durchführung

□ monozentrisch/multizentrisch

Multizentrisch: Aachen und Essen

□ **Verfahren zur Aufklärung und Einholung der Einwilligung**

Die Studienteilnehmer werden mündlich entweder im persönlichen Kontakt, über Telefon oder Videokontakt über die Eckpunkte der Studienteilnahme informiert und erhalten die Studieninformation und die Einverständniserklärung ausgedrückt oder per e-mail. Frühestens einen Tag später werden die Interessenten zur ersten Eingangsuntersuchung persönlich gesehen und nach Klärung von Rückfragen und Unterschrift unter die Einverständniserklärung können die ersten studienbezogenen Maßnahmen nach Protokoll beginnen.

□ **Beschreibung der Datenquellen (Krankenakten, Fragebögen etc.)**

Das primäre Zielkriterium basiert auf EEG-Daten. Dies umfasst die Rohdaten und Daten, die in der Auswertung entstehen. Im Weiteren werden Daten aus Fragebögen, Ratings im Rahmen der Beurteilung von klinischer Symptomatik und Verhaltensdaten im Rahmen der Kognitionsuntersuchung erhoben. Bei Frauen: Speichelprobe zur Hormonbestimmung.

□ **Auflistung/Beschreibung der zu erfassenden Daten**

2.3.2. **Clinical assessments**

All participants will be investigated clinically using the Mini-International-Neuropsychiatric Interview (M.I.N.I., Sheehan et al., 1998) to document and verify the clinical diagnoses. Moreover, all participants will be examined using standardized ratings and questionnaires for psychiatric symptoms (Montgomery Asberg Rating Scale (MADRS, Montgomery & Asberg, 1979), Hamilton Depression Rating scale (HDRS), Clinical Global Impression (CGI), Beck Depression Inventory-II (Beck, Steer, Ball, & Ranieri, 1996). Moreover, we will assess anxiety using the State-Trait Anxiety Inventory (STAI)(Spielberger, Gorsuch, & Lushene, 1970). Cognition (Lee, Hermens, Porter, & Redoblado-Hodge, 2012) will be assessed by a neuropsychological assessment comprising a word knowledge test (MWT-B, Lehrl, 2005), memory (Auditory Verbal Learning Task (AVLT), (Helmstaedter & Durwen, 1990) a German language equivalent to the Rey (1964) AVLT, Trail Making Test Part A/B (Army Individual Test Battery (1944)),

verbal fluency (Controlled Oral Word Association Task, using letters F,A,S,(Benton & Hamsher, 1989)), Digit Symbol Substitution Task (Jaeger, 2018), digit span forward and backward (WAIS, Wechsler, 2008), spatial span forward and backward (Corsi, 1972) and cognitive flexibility as measured with the Nelson (1976) version of the Wisconsin Card Sorting Test. Cognition will be used to assess its impact on EEG and ERP data and on bias measures in secondary analyses.

Current general medical conditions will be assessed by the 14-item Cumulative Illness Rating Scale (CIRS)(Linn, Linn, & Gurel, 1968; Salvi et al., 2008) and occupational and interpersonal adjustment with the Work and Social Adjustment Scale (Mundt, Marks, Shear, & Greist, 2002). Childhood maltreatment experience will be assessed using the CTQ (Bernstein et al., 2003) and impulsivity with the Barratt Impulsivity Scale (BIS-11) (Patton, Stanford, & Barratt, 1995).

While the primary study outcome will be change in EPN amplitude, primary clinical outcome will be the pre/post, change in MADRS scores. Secondary clinical outcome measures will be the change in HDRS rating and other questionnaires. From the first to the last On-Site visit, the assessments will take about 9 weeks including 4 On-Site visits and a 4-week follow-up. The CBM training will start with the pre-intervention EEG assessment. In the second week of the training phase, participants will be contacted by phone or video in order to enhance training compliance. With the post-EEG assessment, tablets will be recollected and training data will be transferred and added to the study database. Following the follow-up assessment, participants will be debriefed and receive their study allowance of 100 €. Clinical data on the primary outcome will be assessed at visits V3 (pre), V5 (post) and V6 (FU). Data from Visit V2 will serve for subject characteristics and for variables which are not followed up over time in the study here, among them trauma experiences (CTQ), cognition and impulsivity scales (BIS-11).

2.3.3. CBM training and task

Allocation to the experimental and the control training conditions in the CBM positivity training will be done randomly as indicated above. The CBM positivity training follows our previous work (Vrijsen et al., 2018). It utilizes a Dot-Probe task that uses 100 positive and 100 neutral pictures from the International Affective Picture System (IAPS, Lang, Bradley, & Cuthbert, 1997) covering a broad range of content categories (e.g., animals, people, objects; in approximate equal proportions). Following a fixation cross in the middle of the display for 1000 ms, two pictures with one neutral and one positive valence, appear above and below the fixation cross for 500ms. Positions for neutral and positive valence pictures are randomly selected. With disappearance of the stimuli, an arrow occurs pointing either to the right or left at one of the positions of the previously shown pictures. According to the direction of the arrow, the subject has to tap to the part of the smartphone screen. Reaction times i.e. the delay between arrow appearance and the subject's reaction are recorded. Following the reaction, a feedback showing either a green check or a red cross indicates success or an error for 500 ms. While in the Vrijsen et al. (2018) study we applied only negative (error) feedback, here we give feedback for both feedback conditions in order not to focus on the negative outcomes with the feedback signal. We present both a bit shorter with 500 ms instead of 1000 ms, in order to facilitate work-flow.

Session structure includes eight practice trials and 240 training trials. The first, the 6th and the 10th session is preceded by pre-training trials using 10 trials in each condition with the probe (arrow) at the positive or the neutral position and arrows pointing to left or right. These 40 trials at the start, the mid-session and at the end of the training are used to calculate the positivity bias in participants and to monitor it's change with training sessions as a form of internal control condition. In the **positivity training** trials, the probe (arrow) always appears at the position of the positive stimulus, thereby implicitly training the subject to direct attention to the positive stimuli. In the **control training condition**, the probe (arrow) is randomly switched between previous positive and neutral valence pictures with 50% probability. No more than three trials of the same type are presented successively in the control condition. Once started, the training runs automatically for forty trials, after which the subject has to press a "continue" button in order to initiate the next forty trials.

Figure 3: CBM app implementing the dot-probe task

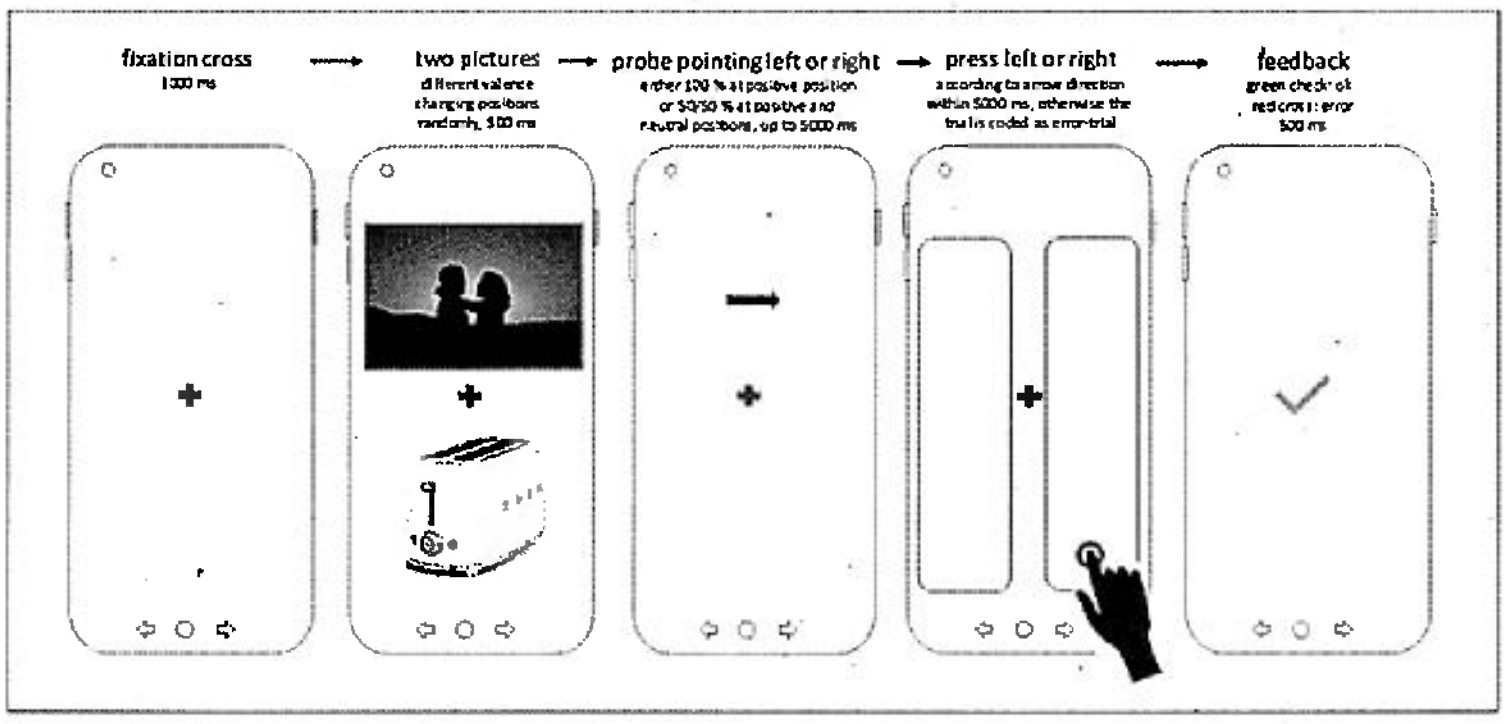

Experimental data are recorded offline on the smartphone using only pseudonymized data and are transferred via the Presentation mobile system. Data are stored using the participants study-number without storing names or other personal data of the participant. Figure 3 demonstrates the schematic implementation of the app. The programming is implemented using Presentation mobile software (Neurobehavioral Systems Inc.). A proof of principle can be found implemented in the "Psych Lab 101" app on Android and Apple systems. While the Presentation mobile app is new, we apply for professional help with experiment coding in order to ensure a stable and bug-free application use within the study.

2.3.4 Resting state EEG

For resting state EEG, continuous EEG data will be recorded for 5 minutes with eyes open and additional 5 minutes eyes closed. The order of resting state EEG followed by emotion processing task to emotion induction task will be the same across all subjects and measurement occasions. Recording resting state data first will leave subjects without recording dependent mood inductions. Emotion processing is suggested to induce less long-lasting emotion change as compared to the mood induction condition and the mood induction task will therefore be conducted as the last part of the assessment.

2.3.5 Emotion processing task

The emotion induction task is designed to assess evoked potentials related to emotion processing derived from EEG. From the IAPS picture set (Lang et al., 1997) pictures with neutral and high and low positive and negative valence will be used. These will be presented on screen with a stimulus onset asynchrony of 2.5 seconds and a picture presentation time of 0.5 seconds for each picture. A fixation cross will be presented in the inter-trial interval. Pictures will be presented embedded in either blue or red frames. Upon the appearance of the red frame the subject is asked to press a button with the right index finger. 50 % stimuli will be non-target neutral pictures of the remaining 50% target stimuli, 10% (per condition) will be accompanied by neutral, high or low arousal positive or high or low arousal negative stimuli. The emotion processing task is designed to last 30 minutes with one planned break in between. The task is intended to evoke occipital P100, N170 and early processing negativity (EPN) potentials. Target stimuli will evoke a P300-like component, which will allow to assess the early, middle and late LPP from 400-600 ms, 600-800 ms and 800-1000 ms latency from sensors around electrodes Fz, Cz and Pz.

2.3.7 EEG data acquisition

EEG Data will be recorded in a dedicated EEG recording room using Brain Products BrainAmp (DC) amplifiers and 64 Ag/AgCl ActiCap Electrodes in Essen as well as in Aachen. Data will be sampled at 1kHz and recorded in a frequency range of 0.03 to 250 Hz. EEG Data will be referenced against

electrode Fz and grounded at Fpz. With offline analysis EEG data will be reafferenced to linked earlobe electrodes. An electrode below the right eye will provide data for vertical eye movement and two electrodes at the outer canthi of both eyes will serve horizontal eye movement correction. ActiCap electrodes provide improved electronic shielding at the electrode resulting in lowered artifacts. Subjects will be seated in a comfortable chair to minimize subject movement throughout the EEG assessment. Data will be stored using Brain Products Recorder software. Visual stimulation will be controlled by Presentation Software (Neurobehavioral Systems Inc.) and visual stimuli will be presented via an LCD monitor at 25 cm height, located 2 meters from the subject's eye resulting in a viewing angle of 6.3°. Subject's responses will be captured by right hand index finger clicks with the mouse connected to the experimental stimulation computer.

2.3.8 Analysis of emotion processing EEG-data

EEG data reafferenced to linked earlobes will be filtered offline between 0.03 and 30 Hz using an 8-order finite impulse response filter. Resulting data will be inspected visually for large or complex artifacts. These will be discarded from further analysis. Horizontal and vertical eye movements and blinks will be removed by independent component analysis as implemented in Vision Analyzer Software 2.1 (Brain Products Inc.). Data will be segmented according to stimulus conditions. These include neutral target and non-target stimuli and target and non-target low and high arousal negative valence stimuli and target and non-target low and high arousal positive valence stimuli. Following baseline correction, resulting segments will be analyzed for remaining artifacts and segments with voltage changes exceeding (80 µV) will be discarded from further analysis. Single subject averages will be computed for the neutral and each of the four combinations of arousal and valence. From these single subject averages, we will derive the EPN mean amplitude between 230 ms and 290 ms post stimulus and P100 and N170 amplitudes and latencies by peak detection at occipital electrodes. Additionally, we will determine P300 amplitudes and latencies at midline electrodes from Fz to Pz and assess the late positive component between 400 ms and 600 ms from electrodes electrodes Pz and around Pz (Schindler & Straube, 2020) and later between 600 – 800 ms and 800 – 1000 ms for the middle and late LPP (Hajcak et al., 2010).

2.3.10 Resting state data analysis

Microstate analysis will be performed using CARTOOL (brainmapping. unige.ch/cartool, Denis Brunet). For each participant, we will calculate the global mean field power (GFP) across all electrodes. For microstate segmentation, the data will be downsampled by only including points where the GFP have a local maximum. Microstate transitions preferentially occur at GFP local minima and this downsampling does not significantly impact microstate segmentation results (Brunet, Murray, & Michel, 2011; Michel & Brunet, 2019; M. M. Murray, Brunet, & Michel, 2008). The data will then be spatially filtered to enhance signal-to-noise ratio (Brunet et al., 2011; E. A. Murray, Wise, & Drevets, 2011; M. M. Murray et al., 2008). Next, the data will be run through a series of polarity-insensitive k-means clustering analyses with k ranging from 1 to 12. A meta-criterion based on taking the median value of six distinct functions will be used to determine the optimal number of microstates (Murphy et al., 2020). We subsequently will run a second round of clustering on the pooled microstates for subjects (Brunet et al., 2011; E. A. Murray et al., 2011; M. M. Murray et al., 2008) within the control and MDD groups. The meta-criterion will be used to determine the optimal number of microstates. For microstate fitting, for each time point in the original data, we will choose the microstate map from the subject's group that most closely resembles the voltage topography insensitive to polarity. Fits will be smoothed on 3 sample half-windows with Besag factor 10 (Murphy et al., 2020). We will obtain a sequence of microstate labels with the same length as the original data set. For each record and microstate, we will calculate three parameters. Proportion will be the amount of the record covered by each microstate, duration will be the average length of time a microstate lasts, and occurrence will be the number of times that microstate occur per second. For the fitted microstate sequences, we calculate a Markov matrix on the observed transitions between microstates (Murphy et al., 2020). We

will calculate an expected Markov matrix by computing the expected transition probabilities given the distribution of microstate labels.

2.3.11 Hormonal cycle

Hormonal cycle and contraceptiva in women will be assessed both clinically and by saliva hormone and metabolite measurements in order to be able to consider this information as possible confounder in the analyses. (Andreano & Cahill, 2010; Arelin et al., 2015; Sundstrom Poromaa & Gingnell, 2014).

▯ Zeitlicher Ablauf für den einzelnen Studienteilnehmer:

In patients, the CBM intervention will be done while continuing their outpatient treatments including pharmacotherapy and psychotherapeutic interventions without interference by the study team thereby continuing TAU in outpatients. Subjects professional contacts with physicians and therapists between pre-training assessments and follow-up will be registered in order to control TAU intervention intensity throughout study participation.

Patients as well as controls will be pre-investigated per phone-call as a screening assessment (V1, day -7). The first on-site visit will start with signing the informed consent sheet and clinical assessments, cognitive testing (V2, day -1), saliva sample and EEG/ERP baseline assessment and depression ratings (V3, day 0). Then, participants will train the CBM task on a dedicated tablet for two weeks. During the two weeks of training they will be contacted by phone in order to ensure adherence and to rule out any technical issues (V4, day 7). Participants will be re-investigated on-site at day 14 (V5) for clinical and EEG assessments. In order to describe the intermediate course of the disease in particular with regards to research question a follow-up visit assessing clinical symptoms will be done at day 42 (V6), resulting in a four-week follow-up period. Specific clinical assessments (i.e. MINI) and questionnaires covering past experiences (i.e. CTQ) and the cognition assessment will be confined to the initial pre-intervention assessments. Study flow is outlined in Figure 2.

Figure 2: Trial flow scheme

Visit<br>Day	V1 Screening<br>-7	V2 Start<br>-1	V3 EEG prae<br>0	V4 Support<br>7	V5 EEG post<br>14	V6 FU<br>42
Contact<br>Work programme	Telephone<br>Screening & Study Information	On Site<br>Consent<br>MINI + cognition,<br>CIRS/CTQ/BIS-11	On Site<br>Prae-EEG<br>MADRS/HAMD<br>BDI/CGI<br>Tablet out	Telephone<br>Support + Motivation	On Site<br>Post-EEG<br>MADRS/HAMD<br>BDI/CGI<br>Tablet back	On Site<br>MADRS/HAMD<br>BDI/CGI<br>Debriefing<br>Allowance
CBM Training				10 CBM positivity tablet trainings in two weeks between day 0 and day 14		

▯ Voraussichtliches Ende der Datenerfassung

2 Jahre nach Projektstart.

9. Nutzen-Risiko-Abwägung

▯ mit der Studienteilnahme verbundener individueller Nutzen

- Eigennutzen (für die teilnehmenden Personen selbst)
  Für die Patienten ist in der aktiven Behandlungsgruppe eine leichte Verbesserung der depressiven Symptomatik zu erwarten. Für die Patienten, die an dem Kontrolltraining teilnehmen wird ebenfalls eine Verbesserung der Stimmung in geringerem Umfang im Rahmen ihres „Treatment As Usual" erwartet. Bei Kontrollprobanden wird erwartet das sich keine spezifische Veränderung in Bezug auf depressive Symptomatik ergibt. Diese Annahmen ergeben sich aus den Vorarbeiten und Vorarbeiten anderer, die nicht-klinische Gruppen untersucht haben (s. Studienprotokoll).

- × Gruppennutzen (zukünftig für gleich erkrankte Personen)
  Die Studie soll das Verständnis über automatisierte Verarbeitungsprozesse insbesondere Positiver Stimuli bei Menschen mit Depression erhöhen. Wenn die Hypothesen bestätigt werden, werden sie einen kleinen Beitrag dazu leisten, dass die Bedeutung der automatisierten Verarbeitung spezifisch auch positiver Stimuli ein relevantes Ziel in der Behandlung von Major Depression darstellt.
- × Fremdnutzen (für die Heilkunde oder Wissenschaft)
  Der oben beschriebene Nutzen ist dementsprechend auch einer für mögliche neue wissenschaftlich fundierte Behandlungsansätze bei Depressionen.
- Wie ist der Nutzen bestimmt worden? (Methode, Kriterien, Literatur)

  Über Literatur und Vorarbeiten. Die Details zur Literatur finden sich im Studienprotokoll. Dort wir auch die studienspezifische Vorarbeit beschrieben.

? mit der Studienteilnahme verbundene Belastungen und Risiken

- Welcher Art sind die möglichen Risiken, Beeinträchtigungen, Belastungen oder sonstigen Nachteile für die teilnehmende Person?

  EEG-Untersuchungen können bei der Montage der Hauben und der Verminderung der Übergangswiderstände ggf. leichte Hautiritationen entstehen. Mit den hier zu verwendeten Acti-Cap Elektroden (Brain Products) reichen 50 kOhm Übergangswiderstand. Die Wahrscheinlichkeit für eine entsprechende Hautirritation ist vor diesem Hintergrund gegenüber den früher gebräuchlichen EEG-Systemen sehr gering. Die übrigen studienbezogenen Methoden (strukturierte Interviews zur klinischen Symptomatik, kurze neuropsychologische Untersuchung mit dem Syndrom-Kurz-Test, Fragebögen zu psychologischen Dimensionen von Angst, Depression und Impulsivität und die Dot-Probe Task basierten Aufgaben des Positivitätstrainings sind nicht-invasiv und mit keinen Nebenwirkungen verbunden.
- Wie ist das Risiko bestimmt worden? (Methode, Kriterien, Literatur)

  Alle hier benannten Methoden entsprechen klinischen Routine-Untersuchungen. Das Positivitätstraining wurde in einer vorauslaufenden Untersuchung unter Verwendung von stationären Computern ohne Nebenwirkungen durchgeführt.
- Inwieweit weichen studienbezogene Maßnahmen von der üblichen Routinebehandlung ab? Alle Verfahren, einschließlich der EEG-Untersuchung entsprechen Standardverfahren klinischer Untersuchungen und Behandlungen.
- Kommt es im Rahmen der Studiendurchführung zu einer Strahlenanwendung?

  × nein

? Statement zur ärztlichen Vertretbarkeit

Die möglichen Hautirritationen bei der EEG-Untersuchung sind selten und verursachen vorübergehendes Unwohlsein, das der Nutzen durch den Erkenntnisgewinn, der Studie aufgewogen wird.

10. Biometrie

? Fallzahlschätzung anhand der primären Zielgröße und der relevanten Effektstärke

Übernahme aus DFG-Antrag (in Englisch):

For the EPN as main ERP-related outcome variable we will calculate mean amplitudes between 230 and 280 ms as indicated by our paradigm specific preliminary data. From mean amplitude EPNs to neutral standard and to each of the four emotion conditions (valence how/high, arousal low/high) we

calculate difference scores subtracting standard from emotion conditions. This yields EPN difference values indicating a negative amplitude change at an occipital sensor combining occipital electrodes. In our preliminary data, we found EPN amplitude related evoked potential changes induced by the emotion conditions related to neutral standard stimuli of -1.46 µV with an SD of 1.7 µV.

For power calculation, we use the high positive condition and for effect size determination we assume a small unspecific effect in the clinical control condition training group of -0.2 µV (SD=1.7). In the clinical positivity training group, we assume an additional training effect of 1.0 µV resulting in a pre/post positivity training EPN change of -1.2 µV (SD=1,7) among patients in the positivity training). Using G*Power (V3.1.9.7) this results in an effect size of d = 0.58.

Given alpha = .05 and 1-beta = 0.8, in a two-tailed t-test using treatment related (pre/post) difference scores in two independent groups (patients with positivity training (-1.2 µV) vs. patients with control training (-0.2 µV)) a total sample size of 94 (= 2 x 47) subjects is needed to reach a critical t of 1.99 as indicated by G*Power (V3.1.9.7). Considering drop-outs due to technical problems as well as patient compliance, an initial sample size of 120 patients will be sufficient to detect treatment related changes in emotion processing as indicated by the EPN component in the processing of high arousal positive valence stimuli.

Considering control subjects, we have no directed hypothesis on the effect of the training on the EPN in controls when compared to the control condition in controls. There may be a small effect in controls, which however is suggested to be of no clinical relevance. When we suggest such a small effect of the positivity training on the EPN of -0.2 µV and an SD of 1.7 in the experimental control group to compare this with the experimental patient group, this will result in similar power estimates as described above in order to demonstrate that the positivity training in patients is specifically effective to change processing of emotion stimuli as compared to the positivity training condition in controls. Thus, we need 60 healthy controls in the experimental group and therefore in total 120 healthy controls.

▯ Geplante statistische Testverfahren

The primary endpoint will be assessed as described in the power-calculation with two-tailed t-tests for independent groups on difference scores on the EPN in the high arousal positive valence condition between the patient experimental and patient control group and between the patient experimental and the control experimental positivity training group.

For analysis of baseline differences an analysis of covariance (ANCOVA) with age and gender as covariates will be used to explore differences between patients and controls on EPN amplitudes (objective 1), as well as LPP amplitudes (objective 3) and occurrence and duration of microstate D (objective 5). Moreover, differences in alpha activity over the frontal lobe and decreased alpha 1 and beta 2 activity over the parietal lobe will be also explored using ANCOVA (objective 5). For additional analyses of treatment effects across the factorial model of high and low arousal and positive and negative valences, we will use a repeated measurement analysis of covariance (ANCOVA) to assess the interactive effects of time (before and after training), group (patients in positivity training, patients in control condition), stimulus arousal and valence on the EPN amplitudes (objective 2). Moreover, a similar statistical design will be used to investigate the difference between patients in positivity training and healthy controls in positivity training (objective 2). This procedure will further be used for changes in behavioural measures (such as reaction times) see clinical objective 3). With respect to amplitudes and latencies of LPP (objective 4) and duration and occurrence of microstate D (objective 6) the same statistical design will be used.

Change in amplitudes of EPN will be correlated with MADRS scores using Spearman' s correlations (objective 2). Pearson correlations will be used to analysis the association between microstates D and EPN amplitudes (objective 5). Spearman correlations will be used to explore associations between childhood adversity and neurophysiological measures and to determine associations between cognitive status and neurophysiological measures (objective 7). Gender effect will be explored in the above mentioned ANCOVA designs. Pearson correlations will be used to investigate the association between hormonal status and neurophysiological measures in females. (all objective 7).

To explore clinical effects of the positivity training on depression severity measured by MADRS ART analysis for repeated measurements will be used given the non-parametric scale of the MADRS scores (Wobbrock, Findlater, Gergle, & Higgins, 2011).

Secondary analyses for which we did not formulate directed hypotheses will be exploratory, in order to further understand the effects of probable confounding variables like past life experiences, clinical symptom expressions, gender, hormonal status and cognition may have on emotion processing and brain states in the application of the positivity training

11. Datenmanagement und Datenschutz

 Verantwortliche/r für die Datenverarbeitung; Datenschutzbeauftragte/r von Initiator und Studienzentrums[5]

Klinik für Psychiatrie, Psychotherapie und Psychosomatik, Universitätsklinikum Aachen:

Die Verarbeitung und Aufbewahrung der Daten im Standort Aachen wird verantwortet vom Studienleiter Herrn Prof. Dr. med. Thomas Frodl, Direktor der Klinik für Psychiatrie, Psychotherapie und Psychosomatik. Datenschutzbeauftragter der Klinik für Psychiatrie, Psychotherapie und Psychosomatik ist PD Dr. Tanja Veselinovic und Datenschutzbeauftragter im Universitätsklinikum Aachen ist Joachim Josef Willems.

LVR-Klinikum Essen:

Die Verarbeitung und Aufbewahrung der Daten wird verantwortet vom lokalen Studienleiter PD. Dr. phil. Bernhard Müller, LVR-Klinikum Essen, Virchowstraße 174, 45147 Essen.

Zuständiger Datenschutzbeauftragter ist Herr John Büder, Landschaftsverband Rheinland (LVR), Telefon: 0221 809-2550, E-Mail: datenschutzbeauftragter@lvr.de. Die Genehmigung des Datenschutzbeauftragten des LVR-Klinikum Essen ist angefragt und wird nachgereicht.

[5] Verantwortlicher für die Datenverarbeitung ist zumindest auch der Initiator (Sponsor oder sonstiger Träger), weil dieser – gegebenenfalls gemeinsam mit anderen – über Zwecke und Mittel der Datenverarbeitung entschei-det (vgl. die Definition in Art. 4 Nr. 7 DSGVO). Deshalb ist hier zunächst der Initiator und dessen Datenschutzbe-auftragter zu nennen. Daneben kommt auch eine Verantwortlichkeit des lokalen Studienzentrums in Betracht.

▯ Erfassung, Speicherung (Art, Ort, Dauer) und Weitergabe von Daten, Gewährleistung der Datensicherheit

- Welche Daten werden erfasst?

  Es werden klinische Daten zu Symptomausprägungen aus Interviews und Fragebögen erfasst. Es werden Kognitionsdaten einer kurzen neuropsychologischen Untersuchung erfasst, es werden EEG-Daten erfasst und nur bei Frauen Hormonspiegel von Geschlechtshormonen aus einer Speichelprobe erfasst. Es werden Trainingsdaten (Reaktionszeiten in der Dot-Probe Task) erfasst.

- In welcher Form, wie lange und wo werden diese gespeichert?

  Einige Daten werden in Papierform vorliegen (Fragebögen, klinische Symptomratings. Andere Daten (EEG und Trainingsdaten) liegen elektronisch vor. Die papierbasierten Daten werden in einer projektbezogenen Datenmatrix des Statistikprogramms SPSS auf Forschungscomputern und zusätzlich auf separaten USB-Festplatten gespeichert und gesichert. Alle diese Daten (elektronisch und papier-basiert) werden pseudonymisiert mit Studiencodes geführt. Eine Verschlüsselungsliste, bei der auch die Einverständniserklärungen der Teilnehmer geführt werden, wird separat in einem verschlossenen Schrank geführt.

- Wer hat Zugriff auf die Daten?

  Auf die Entschlüsselungstabelle haben die Studienleiter und die aufklärenden Studienmitarbeiter Zugriff. Die pseudonymisierten Studienunterlagen werden abgeschlossen gelagert und Studienmitarbeiter und Mitglieder der Forschungsgruppen der Studienleiter haben darauf Zugriff (d.h. Projektmitarbeiter und stud. Hilfskräfte oder Labormitarbeiter unter der Aufsicht der Studienleiter). Das gleiche gilt für die elektronischen Daten, diese werden auf dezidierten Rechnern gespeichert, die passwortgeschützt in Forschungsräumen der Klinik stehen und auf die nur Mitarbeiter der Arbeitsgruppe, aber kein sonstiges Personal Zugriff haben.

- Erfolgt eine Datenweitergabe? Wenn ja, an wen, in welcher Form und zu welchem Zweck?

  Die pseudonymisierten und digitalisierten Daten werden zur gemeinsamen Auswertung zwischen den Standorten Aachen und Essen ausgetauscht. Die Entschlüsselungslisten bleiben im jeweiligen Zentrum und verlassen die jeweilige Klinik nicht. Die Speichelproben werden nach Aachen transferiert und dort analysiert. Probenreste werden nach der Analyse vernichtet.

- Liegt eine Genehmigung / Zustimmung des UME-Datenschutzbeauftragten zum Datenschutzkonzept vor?

  Die Genehmigung des Datenschutzbeauftragten des LVR-Klinikums Essen ist und des Universitätsklinikum Aachen ist angefragt und wird nachgereicht.

▯ anonymisiert/pseudonymisiert

Die Daten werden pseudonymisiert mit Schlüsseltabellen in den jeweiligen Standorten Aachen und Essen erhoben und zu Analysezwecken pseudonymisiert ausgetauscht.

Mit Abschluss der Daten-Erhebung – etwa zwei Jahre nach Einschluss des ersten Teilnehmers - werden die elektronischen Daten anonymisiert (durch Umkodierung des Studienkürzels in eine laufende Nummer, Entfernung von Monat und Tag aus dem Geburtsdatum und Entfernung von datumsbezogenen Variablen aus den elektronischen Daten (z.B.

Datumsangaben zu Studienuntersuchungen). Diese anonymisierten Daten sollen dann einem Datenrepository zugeführt werden um die Daten einer weiteren Verwertung im Sinn einer Nachhaltigkeit und Replizierbarkeit in der Forschung zur Verfügung zu stellen. Wenn die Universitäten Duisburg-Essen und Aachen bis dahin kein eigenes Repository zur Verfügung stellen sollten, sollen die Studiendaten in das Repository für Daten aus dem Gebiet der Psychologie „PsychArchives" des Leibnitz-Instituts für Psychologie überführt werden (https://www.psycharchives.org/).

▯ Widerruf, Datenlöschung

Die pseudonymisierten Quell-Daten verbleiben für 10 Jahre nach Studienende in den Standorten Aachen und Essen und können bis zu 10 Jahre nach Studienende bei Bitte eines Teilnehmers um Löschung elektronisch und physikalisch vernichtet werden.

▯ Rechte auf Auskunft (einschließlich Recht auf kostenfreie Kopie der Daten), Berichtigung, Löschung und Einschränkung der Datenverarbeitung nach der DSGVO etc.[6]

Ein entsprechender Passus ist Teil der Patienteninformation und Einwilligung:

Die Eingabe und Auswertung der Daten in Aachen erfolgt durch die Forschungsgruppe der Klinik für Psychiatrie, Psychotherapie und Psychosomatik, Universitätsklinikum Aachen. Die Verarbeitung und Aufbewahrung der Daten wird verantwortet vom Studienleiter Prof. Dr. med. T. Frodl. Zuständige Datenschutzbeauftragte ist PD Dr. Tanja Veselinovic.

Die Eingabe und Auswertung der Daten in Essen erfolgt durch die Forschungsgruppe der Klinik für Psychiatrie und Psychotherapie, LVR-Klinikum Essen, Klinken der Universität Duisburg-Essen. Die Verarbeitung und Aufbewahrung der Daten Essen wird verantwortet vom lokalen Studienleiter PD. Dr. phil. Bernhard Müller, LVR-Klinikum Essen, Virchowstraße 174, 45147 Essen. Zuständiger Datenschutzbeauftragter ist Herr John Büder, Landschaftsverband Rheinland (LVR), Telefon: 0221 809-2550, E-Mail: datenschutzbeauftragter@lvr.de

Die Teilnehmer*innen haben das Recht, sich bei der Datenschutz-Aufsichtsbehörde zu beschweren: Landesbeauftragter für Datenschutz und Informationsfreiheit Nordrhein-Westfalen, Postfach 20 04 44, 40102 Düsseldorf, Tel.: 0211/38424-0, E-Mail: poststelle@ldi.nrw.de.

Die Teilnehmer*innen haben das Recht, Auskunft über Ihre personenbezogenen Daten zu erhalten, einschließlich der unentgeltlichen Überlassung einer Kopie. Sie haben das Recht, die Berichtigung bzw. Löschung personenbezogener Daten zu verlangen. Ihre Einverständniserklärung bleibt getrennt von den übrigen Befragungsunterlagen.

Bei Widerruf Ihrer Einverständniserklärung werden Ihre Studiendaten, sofern bereits elektronisch erfasst, gelöscht und ihre Fragebogen und Studienunterlagen vernichtet.

Auf Basis der Daten sollen wissenschaftliche Veröffentlichungen erfolgen, in denen alle Teilnehmer vollkommen anonym bleiben. Das heißt, die Ergebnisse werden nur für die Gesamtgruppe der Studienteilnehmer dargestellt, und die Daten von Einzelpersonen sind aus der Darstellung nicht mehr ersichtlich.

▯ Sofern ausnahmsweise einschlägig: Rechtfertigung für Einschränkungen des Rechts auf Löschung [Art. 17 Abs. 3 lit. c) und d) DSGVO; Art. 9 Abs. 2 lit. i) und j) DSGVO] bzw. BDSG oder – sofern

einschlägig – Landesdatenschutzgesetz sowie für sonstige Beschränkungen im Sinne von Art. 89 Abs. 3 DSGVO[7]

Die am Ende der Studie anonymisierten elektronischen Daten sind auch mit hohem Aufwand nicht mehr einzelnen Personen zuzuordnen und können deshalb nach Übergabe an das Repository nicht mehr gelöscht werden.

12. COVID-19:

Hinsichtlich der **COVID-19 Pandemie** halten wir uns an die zu dem jeweiligen Zeitpunkt geltenden Richtlinien im UK Aachen bzw. LVR-Klinik der UK Essen-Duisburg. Derzeit sind dies die AHA-Regeln sowie tragen von FFP2 Masken im direkten Patientenkontakt. Ab 15.3. unterliegen voraussichtlich alle Mitarbeiter der einrichtungsbezogenen Impfpflicht.

13. Unterschriften: Studienleiter/in (Antragsteller/in)

Aachen, 11.02.2022

Prof. Dr. med. T. Frodl

PD Dr. phil. B. Müller

Literatur

Andreano, J. M., & Cahill, L. (2010). Menstrual cycle modulation of medial temporal activity evoked by negative emotion. *Neuroimage, 53*(4), 1286-1293. doi:10.1016/j.neuroimage.2010.07.011

Arelin, K., Mueller, K., Barth, C., Rekkas, P. V., Kratzsch, J., Burmann, I., . . . Sacher, J. (2015). Progesterone mediates brain functional connectivity changes during the menstrual cycle-a pilot resting state MRI study. *Front Neurosci, 9*, 44. doi:10.3389/fnins.2015.00044

Arnold, J. F., Fitzgerald, D. A., Fernandez, G., Rijpkema, M., Rinck, M., Eling, P. A., . . . Tendolkar, I. (2011). Rose or black-coloured glasses? Altered neural processing of positive events during memory formation is a trait marker of depression. *J Affect Disord, 131*(1-3), 214-223. doi:10.1016/j.jad.2010.12.011

Barch, D. M., Whalen, D., Gilbert, K., Kelly, D., Kappenman, E. S., Hajcak, G., & Luby, J. L. (2019). Neural Indicators of Anhedonia: Predictors and Mechanisms of Treatment Change in a Randomized Clinical Trial in Early Childhood Depression. *Biol Psychiatry, 85*(10), 863-871. doi:10.1016/j.biopsych.2018.11.021

Army Individual Test Battery, A. I. T. (1944). Manual of Directions and Scoring. In. Washington DC: War Department, Adjutant General's Office.

Beck, A. T. (1967). *Depression: Clinical, Experimental, and Theoretical Aspects*. New York: Harper & Row.

Beck, A. T. (2008). The evolution of the cognitive model of depression and its neurobiological correlates. *Am J Psychiatry, 165*(8), 969-977. doi:10.1176/appi.ajp.2008.08050721

Beck, A. T., & Haigh, E. A. (2014). Advances in cognitive theory and therapy: the generic cognitive model. *Annu Rev Clin Psychol, 10*, 1-24. doi:10.1146/annurev-clinpsy-032813-153734

Beck, A. T., Steer, R. A., Ball, R., & Ranieri, W. (1996). Comparison of Beck Depression Inventories -IA and -II in psychiatric outpatients. *J Pers Assess, 67*(3), 588-597. doi:10.1207/s15327752jpa6703_13

Becker, E. S., Ferentzi, H., Ferrari, G., Mobius, M., Brugman, S., Custers, J., . . . Rinck, M. (2016). Always Approach the Bright Side of Life: A General Positivity Training Reduces Stress Reactions in Vulnerable Individuals. *Cognit Ther Res, 40*, 57-71. doi:10.1007/s10608-015-9716-2

Benton, A. L., & Hamsher, K. d. (1989). *Multilingual Aphasia Exmination* lova City Iowa AJA Associates.

Bernstein, D. P., Stein, J. A., Newcomb, M. D., Walker, E., Pogge, D., Ahluvalia, T., . . . Zule, W. (2003). Development and validation of a brief screening version of the Childhood Trauma Questionnaire. *Child Abuse Negl, 27*(2), 169-190. doi:10.1016/s0145-2134(02)00541-0

Bondy, E., Stewart, J. G., Hajcak, G., Weinberg, A., Tarlow, N., Mittal, V. A., & Auerbach, R. P. (2018). Emotion processing in female youth: Testing the stability of the late positive potential. *Psychophysiology, 55*(2). doi:10.1111/psyp.12977

Brunet, D., Murray, M. M., & Michel, C. M. (2011). Spatiotemporal analysis of multichannel EEG: CARTOOL. *Comput Intell Neurosci, 2011*, 813870. doi:10.1155/2011/813870

Clark, L. A., & Watson, D. (1991). Tripartite model of anxiety and depression: psychometric evidence and taxonomic implications. *J Abnorm Psychol, 100*(3), 316-336. Retrieved from https://www.ncbi.nlm.nih.gov/pubmed/1918611

Coan, J. A., & Allen, J. J. (2004). Frontal EEG asymmetry as a moderator and mediator of emotion. *Biol Psychol, 67*(1-2), 7-49. doi:10.1016/j.biopsycho.2004.03.002

Corsi, P. M. (1972). *Human memory and the medial temporal region of the brain (Thesis)* Montreal, Canada: McGill University

Cuthbert, B. N., Schupp, H. T., Bradley, M. M., Birbaumer, N., & Lang, P. J. (2000). Brain potentials in affective picture processing: covariation with autonomic arousal and affective report. *Biol Psychol, 52*(2), 95-111. doi:10.1016/s0301-0511(99)00044-7

Dai, Q., Hu, L., & Feng, Z. (2019). Attentional bias modification reduces clinical depression and enhances attention toward happiness. *J Psychiatr Res, 109*, 145-155. doi:10.1016/j.jpsychires.2018.11.024

De Raedt, R., & Koster, E. H. (2010). Understanding vulnerability for depression from a cognitive neuroscience perspective: A reappraisal of attentional factors and a new conceptual framework. *Cogn Affect Behav Neurosci, 10*(1), 50-70. doi:10.3758/CABN.10.1.50

De Vito, D., Al-Aidroos, N., & Fenske, M. J. (2017). Neural evidence that inhibition is linked to the affective devaluation of distractors that match the contents of working memory. *Neuropsychologia, 99*, 259-269. doi:10.1016/j.neuropsychologia.2017.03.022

Frischen, A., Ferrey, A. E., Burt, D. H. R., Pistchik, M., & Fenske, M. J. (2012). The Affective Consequences of Cognitive Inhibition: Devaluation or Neutralization? *Journal of Experimental Psychology-Human Perception and Performance, 38*(1), 169-179. doi:10.1037/a0025981

Golonka, K., Mojsa-Kaja, J., Popiel, K., Marek, T., & Gawlowska, M. (2017). Neurophysiological Markers of Emotion Processing in Burnout Syndrome. *Front Psychol, 8*, 2155. doi:10.3389/fpsyg.2017.02155

Gotlib, I. H., & Joormann, J. (2010). Cognition and depression: current status and future directions. *Annu Rev Clin Psychol, 6*, 285-312. doi:10.1146/annurev.clinpsy.121208.131305

Gotlib, I. H., & McCann, C. D. (1984). Construct accessibility and depression: an examination of cognitive and affective factors. *J Pers Soc Psychol, 47*(2), 427-439. Retrieved from https://www.ncbi.nlm.nih.gov/pubmed/6481620

Gotlib, I. H., Neubauer, D.L. . (2000). Information processing approaches to the study
of cognitive biases in depression. . In S. L. Johnson, Hayes, A.M. (Ed.), *Stress, Coping and Depression* (pp. 117-143). Erlbaum: Mahwah.

Hajcak, G., & Foti, D. (2020). Significance?& Significance! Empirical, methodological, and theoretical connections between the late positive potential and P300 as neural responses to stimulus significance: An integrative review. *Psychophysiology, 57*(7), e13570. doi:10.1111/psyp.13570

Hajcak, G., MacNamara, A., & Olvet, D. M. (2010). Event-related potentials, emotion, and emotion regulation: an integrative review. *Dev Neuropsychol, 35*(2), 129-155. doi:10.1080/87565640903526504

Helmstaedter, C., & Durwen, H. F. (1990). VLMT: Verbaler Lern- und Merkfähigkeitstest: Ein praktikables und differenziertes Instrumentarium zur Prüfung der verbalen Gedächtnisleistungen [VLMT: A useful tool to assess and differentiate verbal memory performance]. *Schweizer Archiv für Neurologie und Psychiatrie, 141*, 21– 30.

Herrmann, M. J., Huter, T., Plichta, M. M., Ehlis, A. C., Alpers, G. W., Muhlberger, A., & Fallgatter, A. J. (2008). Enhancement of activity of the primary visual cortex during processing of emotional stimuli as measured with event-related functional near-infrared spectroscopy and event-related potentials. *Hum Brain Mapp, 29*(1), 28-35. doi:10.1002/hbm.20368

Hill, K. E., South, S. C., Egan, R. P., & Foti, D. (2019). Abnormal emotional reactivity in depression: Contrasting theoretical models using neurophysiological data. *Biol Psychol, 141*, 35-43. doi:10.1016/j.biopsycho.2018.12.011

Jaeger, J. (2018). Digit Symbol Substitution Test: The Case for Sensitivity Over Specificity in Neuropsychological Testing. *J Clin Psychopharmacol, 38*(5), 513-519. doi:10.1097/JCP.0000000000000941

Junghofer, M., Bradley, M. M., Elbert, T. R., & Lang, P. J. (2001). Fleeting images: a new look at early emotion discrimination. *Psychophysiology, 38*(2), 175-178. Retrieved from https://www.ncbi.nlm.nih.gov/pubmed/11347862

https://onlinelibrary.wiley.com/doi/pdfdirect/10.1111/1469-8986.3820175?download=true

Klawohn, J., Burani, K., Bruchnak, A., Santopetro, N., & Hajcak, G. (2020). Reduced neural response to reward and pleasant pictures independently relate to depression. *Psychol Med*, 1-9. doi:10.1017/S0033291719003659

Korb, A. S., Cook, I. A., Hunter, A. M., & Leuchter, A. F. (2008). Brain electrical source differences between depressed subjects and healthy controls. *Brain Topogr, 21*(2), 138-146. doi:10.1007/s10548-008-0070-5

Lang, P. J., Bradley, M. M., & Cuthbert, B. N. (1997). International affective picture system (IAPS): Technical manual and affective ratings. *NIMH Center for the Study of Emotion and Attention*, 39-58.

Lee, R. S., Hermens, D. F., Porter, M. A., & Redoblado-Hodge, M. A. (2012). A meta-analysis of cognitive deficits in first-episode Major Depressive Disorder. *J Affect Disord, 140*(2), 113-124. doi:10.1016/j.jad.2011.10.023

Lehmann, D., & Michel, C. M. (2011). EEG-defined functional microstates as basic building blocks of mental processes. *Clin Neurophysiol, 122*(6), 1073-1074. doi:10.1016/j.clinph.2010.11.003

Lehrl, S. (2005). *Mehrfachwahl-Wortschatz-Intelligenztest (MWT-B)*. Retrieved from Göttingen (Germany):

Levinson, A. R., Speed, B. C., & Hajcak, G. (2019). Neural Response to Pleasant Pictures Moderates Prospective Relationship Between Stress and Depressive Symptoms in Adolescent Girls. *J Clin Child Adolesc Psychol, 48*(4), 643-655. doi:10.1080/15374416.2018.1426004

Linn, B. S., Linn, M. W., & Gurel, L. (1968). Cumulative illness rating scale. *J Am Geriatr Soc, 16*(5), 622-626. doi:10.1111/j.1532-5415.1968.tb02103.x

Macleod, C., & Holmes, E. A. (2012). Cognitive bias modification: an intervention approach worth attending to. *Am J Psychiatry, 169*(2), 118-120. doi:10.1176/appi.ajp.2011.11111682

Menne-Lothmann, C., Viechtbauer, W., Hohn, P., Kasanova, Z., Haller, S. P., Drukker, M., . . . Lau, J. Y. (2014). How to boost positive interpretations? A meta-analysis of the effectiveness of cognitive bias modification for interpretation. *PLoS One, 9*(6), e100925. doi:10.1371/journal.pone.0100925

Michel, C. M., & Brunet, D. (2019). EEG Source Imaging: A Practical Review of the Analysis Steps. *Front Neurol, 10*, 325. doi:10.3389/fneur.2019.00325

Milz, P., Pascual-Marqui, R. D., Achermann, P., Kochi, K., & Faber, P. L. (2017). The EEG microstate topography is predominantly determined by intracortical sources in the alpha band. *Neuroimage, 162*, 353-361. doi:10.1016/j.neuroimage.2017.08.058

Miskowiak, K. W., & Carvalho, A. F. (2014). 'Hot' cognition in major depressive disorder: a systematic review. *CNS Neurol Disord Drug Targets, 13*(10), 1787-1803. Retrieved from https://www.ncbi.nlm.nih.gov/pubmed/25470389

Montgomery, S. A., & Asberg, M. (1979). A new depression scale designed to be sensitive to change. *Br J Psychiatry, 134*, 382-389. doi:10.1192/bjp.134.4.382

Mundt, J. C., Marks, I. M., Shear, M. K., & Greist, J. H. (2002). The Work and Social Adjustment Scale: a simple measure of impairment in functioning. *Br J Psychiatry, 180*, 461-464. doi:10.1192/bjp.180.5.461

Murphy, M., Whitton, A. E., Deccy, S., Ironside, M. L., Rutherford, A., Beltzer, M., . . . Pizzagalli, D. A. (2020). Abnormalities in electroencephalographic microstates are state and trait markers of major depressive disorder. *Neuropsychopharmacology, 45*(12), 2030-2037. doi:10.1038/s41386-020-0749-1

Murray, E. A., Wise, S. P., & Drevets, W. C. (2011). Localization of dysfunction in major depressive disorder: prefrontal cortex and amygdala. *Biol Psychiatry, 69*(12), e43-54. doi:10.1016/j.biopsych.2010.09.041

Murray, M. M., Brunet, D., & Michel, C. M. (2008). Topographic ERP analyses: a step-by-step tutorial review. *Brain Topogr, 20*(4), 249-264. doi:10.1007/s10548-008-0054-5

Nelson, H. E. (1976). A modified card sorting test sensitive to frontal lobe defects. *Cortex, 12*, 313-324.

Patton, J. H., Stanford, M. S., & Barratt, E. S. (1995). Factor structure of the Barratt impulsiveness scale. *J Clin Psychol, 51*(6), 768-774. doi:10.1002/1097-4679(199511)51:6<768::aid-jclp2270510607>3.0.co;2-1

Peckham, A. D., McHugh, R. K., & Otto, M. W. (2010). A meta-analysis of the magnitude of biased attention in depression. *Depress Anxiety, 27*(12), 1135-1142. doi:10.1002/da.20755

Pizzagalli, D. A., Nitschke, J. B., Oakes, T. R., Hendrick, A. M., Horras, K. A., Larson, C. L., . . . Davidson, R. J. (2002). Brain electrical tomography in depression: the importance of symptom severity, anxiety, and melancholic features. *Biol Psychiatry, 52*(2), 73-85. doi:10.1016/s0006-3223(02)01313-6

Polich, J., & Kok, A. (1995). Cognitive and biological determinants of P300: an integrative review. *Biol Psychol, 41*(2), 103-146. doi:10.1016/0301-0511(95)05130-9

Rey, A. (1964). *L'examen clinique en Psychologie*. Paris.

Rinck, M., & Becker, E. S. (2005). A comparison of attentional biases and memory biases in women with social phobia and major depression. *J Abnorm Psychol, 114*(1), 62-74. doi:10.1037/0021-843X.114.1.62

Salvi, F., Miller, M. D., Towers, A. L., Morichi, V., Dessı-Fulgheri, P., Miller, M. D., . . . Dessı-Fulgheri, P. (2008). Guidelines for Scoring the Modified Cumulative Illness Rating Scale. *J Am Geriatr Soc., 56*(10), Appendix S1.

Schindler, S., & Straube, T. (2020). Selective visual attention to emotional pictures: Interactions of task-relevance and emotion are restricted to the late positive potential. *Psychophysiology*, e13585. doi:10.1111/psyp.13585

Schupp, H. T., Flaisch, T., Stockburger, J., & Junghofer, M. (2006). Emotion and attention: event-related brain potential studies. *Prog Brain Res, 156*, 31-51. doi:10.1016/S0079-6123(06)56002-9

Schupp, H. T., Junghofer, M., Weike, A. I., & Hamm, A. O. (2003). Emotional facilitation of sensory processing in the visual cortex. *Psychol Sci, 14*(1), 7-13. doi:10.1111/1467-9280.01411

Schupp, H. T., Schmalzle, R., & Flaisch, T. (2014). Explicit semantic stimulus categorization interferes with implicit emotion processing. *Soc Cogn Affect Neurosci, 9*(11), 1738-1745. doi:10.1093/scan/nst171

Sheehan, D. V., Lecrubier, Y., Sheehan, K. H., Amorim, P., Janavs, J., Weiller, E., . . . Dunbar, G. C. (1998). The Mini-International Neuropsychiatric Interview (M.I.N.I.): the development and validation of a structured diagnostic psychiatric interview for DSM-IV and ICD-10. *J Clin Psychiatry, 59 Suppl 20*, 22-33;quiz 34-57. Retrieved from https://www.ncbi.nlm.nih.gov/pubmed/9881538

http://www.psychiatrist.com/JCP/article/Pages/mini-international-neuropsychiatric-interview-mini.aspx

Sill, J., Popov, T., Schauer, M., & Elbert, T. (2020). Rapid brain responses to affective pictures indicate dimensions of trauma-related psychopathology in adolescents. *Psychophysiology, 57*(1), e13353. doi:10.1111/psyp.13353

Spielberger, C. D., Gorsuch, R. L., & Lushene, R. E. (1970). *Manual for the State-Trait Anxiety Inventory*. Palo Alto Consulting Psychologists Press.

Sundstrom Poromaa, I., & Gingnell, M. (2014). Menstrual cycle influence on cognitive function and emotion processing-from a reproductive perspective. *Front Neurosci, 8*, 380. doi:10.3389/fnins.2014.00380

Sutton, S., Braren, M., Zubin, J., & John, E. R. (1965). Evoked-potential correlates of stimulus uncertainty. *Science, 150*(3700), 1187-1188. doi:10.1126/science.150.3700.1187

Vrijsen, J. N., Fischer, V. S., Muller, B. W., Scherbaum, N., Becker, E. S., Rinck, M., & Tendolkar, I. (2018). Cognitive bias modification as an add-on treatment in clinical depression: Results from a placebo-controlled, single-blinded randomized control trial. *J Affect Disord, 238*, 342-350. doi:10.1016/j.jad.2018.06.025

Vrijsen, J. N., Tendolkar, I., Arias-Vasquez, A., Franke, B., Schene, A. H., Fernandez, G., & van Oostrom, I. (2015). Interaction of the 5-HTTLPR and childhood trauma influences memory bias in healthy individuals. *J Affect Disord, 186*, 83-89. doi:10.1016/j.jad.2015.06.008

Watson, D. (2009). Differentiating the mood and anxiety disorders: a quadripartite model. *Annu Rev Clin Psychol, 5*, 221-247. doi:10.1146/annurev.clinpsy.032408.153510

Wechsler, D. (2008). *Wechsler Adult Intelligence Scale--Fourth Edition. Deutschsprachige Adaptation von F. Petermann*. Frankfurt: Pearson Deutschland GmbH.

Weinberg, A., Hilgard, J., Bartholow, B. D., & Hajcak, G. (2012). Emotional targets: evaluative categorization as a function of context and content. *Int J Psychophysiol, 84*(2), 149-154. doi:10.1016/j.ijpsycho.2012.01.023

Whalen, D. J., Gilbert, K. E., Kelly, D., Hajcak, G., Kappenman, E. S., Luby, J. L., & Barch, D. M. (2020). Preschool-Onset Major Depressive Disorder is Characterized by Electrocortical Deficits in Processing Pleasant Emotional Pictures. *J Abnorm Child Psychol, 48*(1), 91-108. doi:10.1007/s10802-019-00585-8

Winer, E. S., & Salem, T. (2016). Reward devaluation: Dot-probe meta-analytic evidence of avoidance of positive information in depressed persons. *Psychol Bull, 142*(1), 18-78. doi:10.1037/bul0000022

Wobbrock, J. O., Findlater, L., Gergle, D., & Higgins, J. J. (2011). *The Aligned Rank Transform for Nonparametric Factorial Analyses Using Only ANOVA Procedures.* Paper presented at the Proceedings of the ACM Conference on Human Factors in Computing Systems (CHI 2011, Vancouver, British Columbia (May 7-12, 2011).

Xin, W., Yu, R., & Zhao, L. (2021). Event-related-potential based evidence of cognitive dysfunction of processing emotional faces in major depressive disorder patients. *Neurosci Lett, 742*, 135545. doi:10.1016/j.neulet.2020.135545

Aachen, den 14.02.2022

Prof. Dr. Thomas Frodl

Essen, den 13.02.2022

PD Dr. Bernhard Müller
