# Supplementary material for: A tablet-based attentional cognitive bias modification intervention for positive emotion processing in major depressive disorder: a single-blind randomised controlled two-centre trial
Source: eBioMedicine. 2026 Sep 1;132:106453. doi: 10.1016/j.ebiom.2026.106453 (PMC13570274; doi:10.1016/j.ebiom.2026.106453)
Supplement: Supplementary file 3 [file mmc2.docx]

**Caption for Supplementary Material**

**Supplementary File 1 (Appendix Tables)**

Table A1. Means and standard deviations for picture stimuli.

Table A2. Subject characteristics.

Table A3. Type of antidepressants.

Table A4. EPN amplitudes by group, intervention, and time point.

Table A5. Sociodemographic characteristics patients and controls by sex across training conditions

Table A6. Timepoint comparison measures.

Table A7. Linear Mixed Model results for the main effect of time after stratification by depression severity and training group.

# APPENDIX TABLES

Table A1: Means and standard derivation for Picture Stimuli

|  | **Valence** | **Arousal** | **Dominance** |  | **Valence** | **Arousal** | **Dominance** |
| --- | --- | --- | --- | --- | --- | --- | --- |
| **Neutral Picture** | **Mean (SD)** | | | **Positive Picture** | **Mean (SD)** | | |
| 1122 | 1·56 (1·06) | 6·00 (2·46) | 5·86 (2·35) | 1340 | 7·13 (1·57) | 4·75 (2·31) | 5·97 (1·57) |
| 1935 | 4·88 (1·44) | 4·29 (1·95) | 5·71 (1·90) | 1410 | 7·00 (1·46) | 4·17 (2·02) | 3·23 (2·14) |
| 2002 | 4·95 (1·36) | 3·35 (1·87) | 6·10 (2·01) | 1440 | 8·19 (4·61) | 4·61 (2·54) | 3·94 (2·05) |
| 2026 | 4·82 (0·95) | 3·40 (1·84) | 6·42 (1·81) | 1460 | 8·21 (1·21) | 4·31 (2·63) | 3·50 (1·95) |
| 2038 | 5·09 (1·35) | 2·94 (1·93) | 5·95 (1·58) | 1463 | 7·45 (1·76) | 4·79 (2·19) | 3·72 (2·11) |
| 2102 | 5·16 (0·96) | 3·03 (1·87) | 4·87 (1·61) | 1500 | 7·24 (1·88) | 4·12 (2·50) | 3·77 (2·16) |
| 2190 | 4·83 (1·26) | 2·41 (1·80) | 5·51 (1·91) | 1540 | 7·15 (1·96) | 4·54 (2·35) | 6·23 (1·61) |
| 2200 | 4·79 (1·38) | 3·18 (2·17) | 5·92 (2·43) | 1630 | 7·26 (1·48) | 4·45 (2·45) | 2·88 (2·16) |
| 2214 | 5·01 (1·12) | 3·46 (1·97) | 6·11 (2·01) | 1710 | 8·34 (1·12) | 5·41 (2·34) | 3·15 (2·20) |
| 2308 | 5·22 (1·62) | 3·82 (2·15) | 4·22 (2·13) | 1721 | 7·30 (1·39) | 4,53 (2·21) | 5·89 (1·76) |
| 2309 | 4·89 (1·71) | 4·33 (1·92) | 3·48 (2·11) | 1722 | 7·04 (2·02) | 5·22 (2·49) | 5·75 (2·00) |
| 2377 | 5·19 (1·31) | 3·50 (1·95) | 3·98 (2·23) | 1731 | 7·07 (1·58) | 4·56 (2·50) | 5·31 (2·05) |
| 2385 | 5·20 (1·32) | 3·64 (1·81) | 5·95 (1·68) | 1740 | 6·91 (1·38) | 4·27 (2·03 | 5·45 (1·81) |
| 2393 | 4·87 (1·06) | 2·93 (1·88) | 4·17 (2·46) | 1750 | 8·28 (1·07) | 4·10 (2·31) | 5·57 (1·96) |
| 2396 | 4·91 (1·05) | 3·34 (1·83) | 3·47 (1·90) | 1811 | 7·62 (1·59) | 5·12 (2·25) | 5·97 (2·44) |
| 2411 | 5·07 (0·85) | 2·86 (1·84) | 5·81 (2·20) | 1920 | 7·90 (1·48) | 4·27 (2·53) | 5·76 (2·86) |
| 2480 | 4·77 (1·64) | 2·66 (1·78) | 5·81 (2·11) | 1999 | 7·43 (1·47) | 4·77 (2·40) | 7·08 (1·96) |
| 2484 | 5·00 (1·35) | 3·75 (1·90) | 6·44 (2·00) | 2040 | 8·17 (1·60) | 4·64 (2·54) | 6·40 (2·06) |
| 2487 | 5·20 (1·80) | 4·05 (1·92) | 5·96 (1·95) | 2045 | 7·87 (1·19) | 5·47 (2·34) | 6·70 (1·48) |
| 2493 | 4·82 (1·27) | 3·34 (2·10) | 5·53 (2·51) | 2050 | 8·20 (1·31) | 4·57 (2·53) | 5·57 (1·81) |
| 2495 | 5·22 (1·10) | 3·19 (1·76) | 5·78 (1·85) | 2057 | 7·81 (1·28) | 4·54 (2·41) | 3·19 (2·08) |
| 2512 | 4·86 (0·84) | 3·46 (1·75) | 4·99 (2·32) | 2058 | 7·91 (1·26) | 5·09 (2·48) | 4·89 (2·25) |
| 2514 | 5·19 (1·09) | 3·50 (1·81) | 6·25 (1·86) | 2070 | 8·17 (1·46) | 4·51 (2·74) | 5·92 (2·02) |
| 2516 | 4·90 (1·43) | 3·50 (1·88) | 5·20 (2·13) | 2071 | 7·86 (1·32) | 5·00 (2·34) | 5·66 (1·84) |
| 2570 | 4·78 (1·24) | 2·76 (1·92) | 5·96 (1·72) | 2075 | 7·32 (1·65) | 5·27 (2·27) | 6·00 (2·00) |
| 2595 | 4·88 (1·24) | 3·71 (1·88) | 5·23 (2·20) | 2080 | 8·09 (1·47) | 4·70 (2·59) | 3·51 (2·12) |
| 2635 | 5·22 (1·65) | 4·42 (1·98) | 3·85 (1·95) | 2091 | 7·68 (1·43) | 4·51 (2·28) | 3·79 (2·69) |
| 2749 | 5·04 (1·39) | 3·76 (2·03) | 3·79 (2·47) | 2150 | 7·92 (1·59) | 5·00 (2·63) | 6·04 (1·79) |
| 2840 | 4·91 (1·52) | 2·43 (1·82) | 4·74 (2·36) | 2151 | 7·32 (1·63) | 4·37 (2·13) | 3·46 (2·36) |
| 2850 | 5·22 (1·39) | 3·00 (1·94) | 5·31 (2·32) | 2152 | 6·93 (1·42) | 4·50 (2·25) | 6·18 (1·89) |
| 2880 | 5·18 (1·44) | 2·96 (1·94) | 5·85 (1·95) | 2153 | 6·98 (1·50) | 4·40 (2·24) | 6·33 (2·02) |
| 2890 | 4·95 (1·09) | 2·95 (1·87) | 5·83 (1·77) | 2154 | 8·03 (1·13) | 4·48 (2·31) | 5·23 (2·01) |
| 5471 | 5·21 (1·18) | 3·26 (2·05) | 4·22 (2·24) | 2156 | 7·12 (1·46) | 4·34 (2·11) | 3·87 (2·39) |
| 5510 | 5·15 (1·43) | 2·82 (2·18) | 5·89 (2·15) | 2158 | 7·31 (1·48) | 5·00 (2·20) | 3·78 (2·17) |
| 5531 | 5·15 (1·45) | 3·69 (2·11) | 4·37 (2·41) | 2160 | 7·58 (1·69) | 5·16 (2·18) | 2·98 (2·13) |
| 5532 | 5·19 (1·69) | 3·79 (2·20) | 5·92 (1·91) | 2165 | 7·63 (1·48) | 4·55 (2·55) | 5·10 (2·46) |
| 5534 | 4·84 (1·44) | 3·14 (2·03) | 5·18 (2·19) | 2170 | 7·55 (1·42) | 4·08 (2·48) | 6·65 (2·03) |
| 5535 | 4·81 (1·52) | 4·11 (2·31) | 2·66 (2·00) | 2222 | 7·11 (1·54) | 4·08 (2·15) | 4·91 (2·05) |
| 5740 | 5·21 (1·38) | 2·59 (1·99) | 4·88 (2·31) | 2224 | 7·24 (1·58) | 4·85 (2·11) | 6·39 (1·89 |
| 6150 | 5·08 (1·17) | 3·22 (2·02) | 3·45 (1·94) | 2260 | 8·06 (1·42) | 4·26 (2·44) | 6·21 (1·74) |
| 7000 | 5·00 (0·84) | 2·42 (1·79) | 5·91 (1·69) | 2274 | 7·47 (1·51) | 4·22 (2·05) | 6·90 (2·59) |
| 7002 | 4·97 (0·97) | 3·16 (2·00) | 6·64 (1·96) | 2310 | 7·06 (1·52) | 4·16 (2·01) | 2·33 (1·94) |
| 7004 | 5·04 (0·06) | 2·00 (1·66) | 4·00 (2·39) | 2311 | 7·54 (1,37) | 4·42 (2·28) | 3·00 (2·16) |
| 7006 | 4·88 (0·99) | 2·33 (1·67) | 4·85 (2·12) | 2331 | 7·24 (1·72) | 4·30 (2·38) | 5·45 (1·78) |
| 7009 | 4·93 (1·00) | 3·01 (1·97) | 4·62 (1·87) | 2332 | 7·64 (1·60) | 4·30 (2·29) | 5·83 (1·91) |
| 7010 | 4·94 (1·07) | 1·76 (1·48) | 5·93 (1·92) | 2340 | 8·03 (1·26) | 4·90 (2·20) | 6·57 (2·09) |
| 7012 | 4·98 (1·05) | 3·00 (1·94) | 6·40 (2·54) | 2341 | 7·38 (1·59) | 4·11 (2·31) | 6·69 (2·11) |
| 7014 | 5·15 (0·97) | 3·25 (2·03) | 6·07 (1·96) | 2345 | 7·41 (1·72) | 5·42 (2·47) | 6·39 (2·09) |
| 7016 | 4·76 (1·08) | 3·40 (1·71) | 5·99 (1·87) | 2346 | 7·05 (1·53) | 5·28 (2·28) | 6·16 (1·79) |
| 7017 | 5·18 (1·07) | 3·12 (1·97) | 6·23 (1·87) | 2352 | 7·27 (1·79) | 5·16 (2·17) | 6·07 (2·01) |
| 7018 | 4·81 (0·88) | 3·91 (1·97) | 6·49 (2·26) | 2373 | 6·97 (1·32) | 4·50 (2·08) | 2·79 (2·12) |
| 7019 | 5·2 (1·17) | 3·36 (1·87) | 5·89 (1·87) | 2391 | 7·11 (1·77) | 4·63 (2·43) | 6·64 (2·19) |
| 7020 | 4·97 (1·04) | 2·17 (1·71) | 6·16 (1·79) | 2395 | 7·49 (1·69) | 4·19 (2·40) | 3·25 (2·13) |
| 7021 | 5·21 (1·22) | 4·17 (2·22) | 4·72 (1·86) | 2398 | 7·48 (1·32) | 4·74 (2·11) | 6·53 (2·09) |
| 7032 | 4·82 (1·46) | 3·18 (1·88) | 5·86 (2·05) | 2550 | 7·77 (1·43) | 4·68 (2·43) | 5·37 (2·03) |
| 7034 | 4·95 (0·87) | 3·06 (1·95) | 6·04 (2·28) | 2650 | 7·27 (1·67) | 4·28 (2·41) | 4·73 (2·12) |
| 7035 | 4·98 (0·96) | 2·66 (1·82) | 6·28 (2·33) | 2655 | 6·88 (2·09) | 4·57 (2·19) | 5·34 (1·93) |
| 7036 | 4·88 (1·08) | 3·32 (2·04) | 6·23 (2·15) | 2660 | 7·75 (1·48) | 4·44 (2·41) | 4·91 (2·00) |
| 7037 | 4·81 (1·12) | 3·71 (2·08) | 5·03 (2·46) | 4601 | 6·82 (1·22) | 5·08 (2·01) | 4·16 (2·29) |
| 7038 | 4·82 (1·20) | 3·01 (1·96) | 4·59 (2·68) | 4603 | 7·10 (1·58) | 4·89 (2·26) | 3·26 (1·88) |
| 7041 | 4·99 (1·12) | 2·60 (1·78) | 6·33 (1·93) | 4610 | 7·29 (1·74) | 5·19 (2·29) | 5·92 (1·73) |
| 7043 | 5·17 (1·26) | 3·68 (2·09) | 6·45 (2·42) | 4612 | 6·82 (1·67) | 5·06 (1·98) | 5·56 (2·37) |
| 7045 | 4·97 (0·76) | 3·32 (1·96) | 6·43 (2·42) | 4614 | 7·15 (1·44) | 4·67 (2·47) | 5·85 (1·65) |
| 7050 | 4·93 (0·81) | 2·75 (1·80) | 6·00 (1·95) | 4616 | 6·86 (1·52) | 4·43 (2·07) | 6·06 (1·79) |
| 7053 | 5·22 (0·75) | 2·95 (1·91) | 6·09 (1·79) | 4622 | 7· 13 (1·80) | 5·44 (2·23) | 3·60 (2·08) |
| 7055 | 4·90 (0·64) | 3·02 (1·83) | 5·81 (2·09) | 4623 | 5·89 (1·95) | 6·21 (2·26) | 5·97 (2·21) |
| 7056 | 5·07 (1·02) | 3·07 (1·92) | 5·40 (1·58) | 4624 | 6·84 (1·60) | 5·02 (2·05) | 7·08 (1·99) |
| 7059 | 4·93 (0·81) | 2·73 (1·88) | 2·88 (2·14) | 4628 | 7·23 (1·72) | 5·19 (2·11) | 7·03 (2·13) |
| 7077 | 5·12 (1·46) | 4·61 (2·06) | 3·92 (2·28) | 4641 | 7·20 (1·59) | 5·43 (2·10) | 5·90 (2·00) |
| 7090 | 5·19 (1·46) | 2·61 (2·03) | 2·70 (2·02) | 4700 | 6·91 (1·94) | 4·05 (1·90) | 5·86 (1·73) |
| 7100 | 5·24 (1·20) | 2·89 (1·70) | 3·28 (2·15) | 5199 | 6·93 (1·91) | 4·70 (2·52) | 4·58 (2·09) |
| 7130 | 4·77 (1·03) | 3·35 (1·90) | 3·46 (2·07) | 5480 | 7·53 (1·63) | 5·48 (2·35) | 4·98 (2·24) |
| 7160 | 5·02 (1·10) | 3·07 (2·07) | 3·94 (2·55) | 5600 | 7·57 (1·48) | 5·19 (2·70) | 5·68 (2·55) |
| 7161 | 4·98 (1·02) | 2·98 (1·99) | 5·03 (2·18) | 5660 | 6·32 (1·70) | 5·13 (2·06) | 5·03 (1·86) |
| 7170 | 5·14 (1·28) | 3·21 (2·05) | 5·53 (2·01) | 5814 | 7·15 (1·54) | 4·82 (2·40) | 3·38 (2·36) |
| 7175 | 4·87 (1·00) | 1·72 (1·26) | 5·50 (2·46) | 5825 | 8·03 (1·18) | 5·46 (2·72) | 4·14 (2·28) |
| 7179 | 5·06 (1·05) | 2·88 (1·97) | 4·28 (2·56) | 5829 | 7·65 (1·42) | 4·68 (2·45) | 4·43 (2·25) |
| 7182 | 5·16 (1·31) | 4·02 (2·12) | 7·49 (2·39) | 5830 | 8·00 (1·48) | 4·92 (2·65) | 4·31 (2·27) |
| 7184 | 4·84 (1·02) | 3·66 (1·89) | 5·30 (2·21) | 5831 | 7·63 (1·15) | 4·43 (2·49) | 4·22 (2·17) |
| 7185 | 4·97 (0·87) | 2·64 (2·04) | 4·18 (2·27) | 5836 | 7·25 (1·39) | 4·28 (2·41) | 3·86 (2·32) |
| 7187 | 5·07 (1·02) | 2·30 (1·75) | 5·80 (1·77) | 5982 | 7·61 (1·48) | 4·51 (2·85) | 5·52 (2·05) |
| 7207 | 5·15 (1·46) | 3·57 (2·25) | 5·97 (1·89) | 7200 | 7·63 (1·74) | 4·87 (2·59) | 5·44 (2·17) |
| 7211 | 4·81 (1·78) | 4·20 (2·40) | 5·98 (1·69) | 7220 | 6·91 (1·74) | 5·30 (2·35) | 5·57 (1·97) |
| 7217 | 4·82 (0·99) | 2·43 (1·64) | 5·68 (1·96) | 7260 | 7·21 (1·66) | 5·11 (2·19) | 6·00 (1·83) |
| 7233 | 5·09 (1·46) | 2·77 (1·92) | 6·36 (1·85) | 7280 | 7·20 (1·90) | 4·46 (2·38) | 5·85 (1·79) |
| 7235 | 4·96 (1·18) | 2·83 (2·00) | 5·48 (1·90) | 7330 | 7·69 (1·84) | 5·14 (2·58) | 4·87 (2·02) |
| 7247 | 5·05 (1·00) | 4·14 (2·23) | 5·13 (2·45) | 7390 | 6·84 (1·73) | 4·56 (2·28) | 5·77 (2·23) |
| 7248 | 5·22 (1·07) | 4·22 (2·11) | 3·73 (1·88) | 7430 | 7·11 (1·78) | 4·72 (2·29) | 6·27 (2·21) |
| 7249 | 5·24 (1·04) | 3·97 (2·08) | 3·79 (2·01) | 7480 | 7·08 (1·62) | 4·55 (2·42) | 4·39 (2·24) |
| 7255 | 5·07 (1·18) | 3·36 (1·99) | 2·77 (2·24) | 7492 | 7·41 (1·68) | 4·91 (2·46) | 5·81 (2·08) |
| 7287 | 4·77 (1·48) | 3·57 (1·98) | 5·88 (1·87) | 7507 | 6·25 (1·37) | 3·54 (1·95) | 5·37 (2·02) |
| 7365 | 5·20 (1·57) | 4·13 (1·93) | 6·00 (1·67) | 7580 | 7·51 (1·60) | 4·59 (2·72) | 5·37 (2·46) |
| 7476 | 4·99 (2·24) | 4·63 (2·02) | 4·16 (2·02) | 8120 | 7·09 (1·36) | 4·85 (2·13) | 5·91 (2·10) |
| 7484 | 4·99 (1·97) | 4·24 (2·23) | 4·72 (2·21) | 8162 | 6·97 (1·55) | 4·98 (2·25) | 6·39 (1·95) |
| 7487 | 4·92 (1·57) | 4·08 (2·00) | 4·39 (2·06) | 8350 | 7·18 (1·56) | 5·18 (2·28) | 6·36 (1·66) |
| 7491 | 4·82 (1·03) | 2·39 (1·90) | 3·42 (2·31) | 8371 | 6·82 (1·45) | 5·12 (2·30) | 6·14 (2·74) |
| 7547 | 5·21 (0·96) | 3·18 (2·01) | 5·11 (2·23) | 8461 | 7·22 (1·53) | 4·69 (2·20) | 5·89 (2·38) |
| 7705 | 4·77 (1·02) | 2·65 (1·88) | 5·54 (1·95) | 8497 | 7·26 (1·44) | 4·19 (2·18) | 5·15 (2·36) |
| 7950 | 4·94 (1·21) | 2·28 (1·81) | 6·03 (1·86) | 8510 | 7·32 (1·72) | 4·93 (2·56) | 4·23 (2·08) |
| 9070 | 5·01 (1·89) | 3·63 (2·03) | 3·78 (2·15) | 8531 | 7·03 (1·50) | 5·41 (2·15) | 3·90 (2·21) |

Table A2. Subject characteristics.

|  | **Age Mean (SD)** | **PT** | **CT** | **Antidepressant** | **No Antidepressant** | **Woman** | **Man** |
| --- | --- | --- | --- | --- | --- | --- | --- |
| **MDD (n = 119)** | 36·18 (1·176) | 62 | 57 | 72 | 47 | 75 | 44 |
| **HCS (n = 121)** | 33·72 (1·177) | 61 | 60 | ·· | 121 | 86 | 35 |
| **Overall** | 34·94 (12·920) | 123 | 117 | 72 | 168 | 161 | 79 |

*Note. MDD =* Major Depressive Disorder subjects; HCS = healthy control subjects; Values represent means (M) and standard deviations (SD); T = control training; PT = positive training.

Table A3. Type of antidepressants

| **Number of Antidepressants** | **Antidepressant** | **Category** |
| --- | --- | --- |
| 4 | Bupropion | One Medication |
| 1 | Bupropion, Escitalopram | Multiple Medications |
| 1 | Bupropion, Sertraline, Clozapine | Multiple Medications |
| 7 | Citalopram | One Medication |
| 1 | Deferoxamine | One Medication |
| 1 | Elontril | One Medication |
| 1 | Elontril, Mirtazapine | Multiple Medications |
| 13 | Escitalopram | One Medication |
| 3 | Fluoxetine | One Medication |
| 5 | Milnacipran | One Medication |
| 1 | Minaprine, Trazodone | Multiple Medications |
| 1 | Quetiapine | One Medication |
| 1 | St. John‘s Wort | One Medication |
| 13 | Sertraline | One Medication |
| 15 | Venlafaxine | One Medication |

Table A4. EPN amplitudes by group, intervention, and time point.

| EPN data |  | |  |  | |  | |  |  |
| --- | --- | --- | --- | --- | --- | --- | --- | --- | --- |
| Group | Intervention (N) | | Time | Mean (SD) | | 95 % CI | |  |  |
| HCS | CT (50) | | prae | 5·150 (4·82) | | 4·09 - 6·22 | |  |  |
|  |  |  | post | 3·055 (3·13) | | 2·15 - 3·96 | |  |  |
|  | PT (51) | | prae | 3·770 (3·57) | | 2·72 - 4·82 | |  |  |
|  |  |  | post | 3·488 (3·40) | | 2·59 - 4·38 | |  |  |
| MDD | CT (48) | | prae | 3·283 (2·89) | | 2·20 - 4·40 | |  |  |
|  |  |  | post | 3·702 (2·85) | | 2·78 - 4·62 | |  |  |
|  | PT (52) | | prae | 4·048 (3·70) | | 2·99 - 5·10 | |  |  |
|  |  |  | post | 2·820 (3·51) | | 1·93 - 3·71 | |  |  |
|  | |  | | |  | |  | |  |
|  | | | | | | | | |  |

Note: EPN data in µV, mean amplitude, standard deviation (SD) and 95% confidence interval (CI). HCS = healthy control subjects, MDD = major depressive disorder patients, CT = control training, PT = positivity training

Table A5. Sociodemographic characteristics patients and controls by sex across training conditions

5a. Sociodemographic characteristics of patients with major depressive disorder (MDD) by sex across training conditions (N = 119).

|  | **MDD CT Men (n=25)** | **MDD CT Women (n=32)** | **MDD PT Men (n=19)** | **MDD PT Women (n=43)** | **Overall MDD (n=119)** |
| --- | --- | --- | --- | --- | --- |
| **Age** M [SD] Median [Q1; Q3] | 35·54 [12·04] 33·00 [25·75; 43·25] | 34·19 [12·17] 32·50 [24·00; 45·25] | 38·94 [14·35] 32·50 [26·25; 55·25] | 36·91 [13·28] 34·00 [24·00; 51·00] | 36·18 [12·83] 33·00 [24·00; 50·00] |
| **Baseline MADRS** M [SD]  Median [Q1; Q3] | 14·15 [8·37] 15·00 [9·50; 17·50] | 15·84 [9·78] 14·00 [8·00; 22·75] | 14·78 [10·32] 12·00 [8·75; 20·00] | 13·33 [9·11] 12·50 [7·75; 19·25] | 14·42 [9·27] 14·00 [8·00; 20·00] |
| **Baseline HAMD-21** M [SD]  Median [Q1; Q3] | 12·11 [5·92] 13·50 [8·75; 16·00] | 13·22 [7·48] 13·50 [7·00; 19·00] | 10·56 [5·79] 10·50 [6·50; 13·25] | 12·00 [7·62] 13·00 [7·50; 16·25] | 12·14 [6·95] 13·00 [7·00; 16·00] |
| **Baseline BDI-II** M [SD] Median [Q1; Q3] | 21·08 [11·93] 22·00 [10·75; 28·25] | 27·78 [12·44] 30·50 [16·25; 36·75] | 20·61 [10·70] 19·00 [11·50; 29·25] | 22·60 [13·15] 19·00 [12·00; 33·00] | 23·36 [12·52] 23·00 [14·00; 32·00] |
| **Baseline STAI-X2** Median [Q1; Q3] | 55·38 [13·08] 57·00 [44·00; 62·75] | 57·03 [11·93] 60·00 [51·00; 67·00] | 51·17 [9·80] 49·00 [44·50; 59·25] | 56·56 [9·29] 58·00 [49·00; 65·00] | 55·61 [11·05] 55·00 [43·00; 63·00] |
| **Concomitant Treatments**  Yes/No | 17 / 8 | 25 / 7 | 12 / 7 | 36 / 7 | 90 / 29 |
| **Past Psychiatric Treatments** Yes/No | 18 / 7 | 26 / 6 | 16 / 3 | 33 / 10 | 93 / 26 |

*Note.* MDD = major depressive disorder subjects; CT = control training; PT = positive training. Age is reported as mean (M) and standard deviation (SD). BDI-II = Beck Depression Inventory–II; HAMD-21 = 21-item Hamilton Depression Rating Scale; MADRS = Montgomery–Åsberg Depression Rating Scale; STAI-X2 = State- Trait Anxiety Inventory – State Subscale. Values are means (M) and standard deviations (SD)l, with median and interquartile range [Q1; Q3].

Table 5b. Sociodemographic characteristics of healthy control subjects (HCS) stratified by sex and training condition (N = 121).

|  | **HCS CT Men (n = 20)** | **HCS CT Women (n = 40)** | **HCS PT Men (n = 15)** | **HCS PT Women (n = 46)** | **Overall HCS (n = 121)** |
| --- | --- | --- | --- | --- | --- |
| **Age** M [SD] Median [Q1; Q3] | 36·63 [11·04] 35·00 [28·00; 42·00] | 31·10 [10·69] 26·50 [22·00; 40·00] | 37·44 [20·08] 30·00 [25·75; 44·75] | 33·50 [12·25] 29·50 [24·00; 42·25] | 33·72 [12·95] 29·00 [24·00; 42·00] |
| **Baseline MADRS** M [SD] Median [Q1; Q3] | 0·44 [1·04] 0·00 [0·00; 0·25] | 0·80 [2·34] 0·00 [0·00; 1·00] | 0·77 [1·92] 0·00 [0·00; 0·00] | 0·57 [1·87] 0·00 [0·00; 0·00] | ·65 [1·94] ·00 [0·00; 0·00] |
| **Baseline HAMD-21** Median [Q1; Q3] | 0·72 [1·56] 0·00 [0·00; 1·00] | 1·00 [1·59] 0·00 [0·00; 2·00] | 0·85 [1·14] 0·00 [0·00; 1·50] | 0·85 [1·76] 0·00 [0·00; 1·00] | ·88 [1·60] ·00 [0·00; 1·00] |
| **Baseline BDI-II** M [SD] Median [Q1; Q3] | 2·68 [3·50] 1·00 [0·00; 5·00] | 3·85 [4·81] 2·00 [0·00; 6·00] | 3·25 [4·01] 1·00 [0·00; 6·00] | 3·67 [4·70] 2·00 [0·00; 5·25] | 3·52 [4·45] 2·00 [0·00; 6·00] |
| **Baseline STAI-X2** Median [Q1; Q3] | 32·79 [6·65] 31·00 [27·00; 40·00] | 34·28 [11·01] 31·00 [26·00; 43·50] | 35·06 [10·91] 33·00 [24·00; 47·00] | 32·33 [9·55] 31·00 [26·75; 38·00] | 33·41 [9·81] 33·00 [29·00; 42·00] |
| **Concomitant Treatments**  Yes/No | 0 / 20 | 0 / 40 | 1 / 14 | 1 / 45 | 2 / 119 |
| **Past Psychiatric Treatments** Yes/No | 0 / 20 | 1 / 39 | 1 / 14 | 5 / 41 | 7 / 114 |

*Note.* HCS = healthy control subjects; CT = control training; PT = positive training. Age is reported as mean (M) and standard deviation (SD). BDI-II = Beck Depression Inventory–II; HAMD-21 = 21-item Hamilton Depression Rating Scale; MADRS = Montgomery–Åsberg Depression Rating Scale; STAI-X2 = State- Trait Anxiety Inventory – State Subscale. Values are means (M) and standard deviations (SD), with median and interquartile range [Q1; Q3].

Table A6. Timepoint comparison, measures

| **Measure** | **Timepoint comparison** | **Mann-Whitney U** | **Wilcoxon W** | **Test Statistic (z)** | **p-value** |
| --- | --- | --- | --- | --- | --- |
| MADRS | Pre–EEG to Post-EEG | 6382·00 | 19262·00 | ·819 | ·41 |
| MADRS | Post-EEG to Follow-up | 5937·50 | 18817·50 | -·135 | ·89 |
| MADRS | Pre–EEG to Follow-up | 6591·00 | 19471·00 | ·1258 | ·21 |
| HAMD-21 | Pre–EEG to Post–EEG | 6416·00 | 19296·00 | ·873 | ·38 |
| HAMD-21 | Post-EEG to Follow-up | 6158·50 | 19038·50 | ·335 | ·74 |
| HAMD-21 | Pre–EEG to Follow-up | 6507·50 | 19387·50 | 1·066 | ·29 |
| BDI-II | Pre–EEG to Post-EEG | 6299·00 | 19340·00 | -·121 | ·90 |
| BDI-II | Post-EEG to Follow-up | 6759·00 | 19800·00 | ·813 | ·42 |
| BDI-II | Pre–EEG to Follow-up | 6769·50 | 19810·500 | ·818 | ·41 |

*Note.* BDI-II = Beck Depression Inventory–II; HAMD-21 = 21-item Hamilton Depression Rating Scale; MADRS = Montgomery–Åsberg Depression Rating Scale; EEG–pre = electroencephalography recorded before the intervention; EEG–post = electroencephalography recorded after the intervention; follow-up = four weeks after the intervention.

Table A7. Linear Mixed Model Results for main effect time after stratification by depression severity and training group.

| **Severity, Training** | **df** | **F-value** | **p-value** |
| --- | --- | --- | --- |
| *Remitted* |  |  |  |
| MADRS | 1/40·01 | 44·24 | **< ·001**** |
| MADRS - PT | 1/16·52 | 10·62 | **·005*** |
| MADRS - CT | 1/20·01 | 54·02 | **< ·001**** |
| HAMD-21 | 1/105·77 | 176·30 | **< ·001**** |
| HAMD-21 - PT | 1/59·16 | 92·26 | **< ·001**** |
| HAMD-21 - CT | 1/45·11 | 89·31 | **< ·001**** |
| BDI-II | 1/32·94 | 66·12 | **< ·001**** |
| BDI-II -PT | 1/21·82 | 45·76 | **< ·001**** |
| BDI-II -CT | 1/32·94 | 66·12 | **< ·001**** |
| *Minimally Depressed* |  |  |  |
| MADRS | 1/94·61 | 840·83 | **< ·001**** |
| MADRS - PT | 1/52·06 | 466·36 | **< ·001**** |
| MADRS - CT | 1/41·92 | 395·59 | **< ·001**** |
| HAMD-21 | 1/57·35 | 1656·30 | **< ·001**** |
| HAMD-21 - PT | 1/28·61 | 682·44 | **< ·001**** |
| HAMD-21 - CT | 1/27·33 | 943·69 | **< ·001**** |
| BDI-II | 1/13·08 | 1177·66 | **< ·001**** |
| BDI-II -PT | 1/4·73 | 748·75 | **< ·001**** |
| BDI-II -CT | 1/13·08 | 1177·66 | **< ·001**** |
| *Mildly Depressed^X^* |  |  |  |
| BDI-II | 1/20·17 | 1783·25 | **< ·001**** |
| BDI-II -PT | 1/11·53 | 752·55 | **< ·001**** |
| BDI-II -CT | 1/20·17 | 1783·25 | **< ·001**** |
| *Moderately Depressed* |  |  |  |
| MADRS | 1/32·61 | 1054·16 | **< ·001**** |
| MADRS - PT | 1/17·14 | 361·77 | **< ·001**** |
| MADRS - CT | 1/13·56 | 1032·21 | **< ·001**** |
| HAMD-21 | 1/13·14 | 1737·96 | **< ·001**** |
| HAMD-21 - PT | 1/4·69 | 397·00 | **< ·001**** |
| HAMD-21 - CT | 1/7·36 | 1589·90 | **< ·001**** |
| BDI-II | 1/20·94 | 1814·81 | **< ·001**** |
| BDI-II -PT | 1/19·76 | 70·51 | **< ·001**** |
| BDI-II -CT | 1/20·94 | 1814·81 | **< ·001**** |
| *Severely Depressed^xx^* |  |  |  |
| MADRS | 1/5·88 | 362·90 | **< ·001**** |
| MADRS - PT | ·· | ·· | ·· |
| MADRS - CT | ·· | ·· | ·· |
| HAMD-21 | ·· | ·· | ·· |
| HAMD-21 - PT | ·· | ·· | ·· |
| HAMD-21 - CT | ·· | ·· | ·· |
| BDI-II | 1/56·77 | 1287·54 | **< ·001**** |
| BDI-II -PT | 1/27·51 | 796·31 | **< ·001**** |
| BDI-II -CT | 1/56·77 | 1287·54 | **< ·001**** |

*Note.* BDI-II = Beck Depression Inventory–II; HAMD-21 = 21-item Hamilton Depression Rating Scale; MADRS = Montgomery–Åsberg Depression Rating Scale; ^x^ =Mildly depressed severity classification only reported for BDI-II as original scoring instructions for MADRS and HAMD-21 did not include this severity class. ^xx^ = Results only reported for MADRS (overall) and BDI-II subgroups, as other subgroup sample sizes were too small.

# **APPENDIX B**

**Safety monitoring and interim analyses:** Given the non-pharmacological nature of the study (tablet-based intervention and EEG measurements), no formal safety monitoring system or independent data safety monitoring board (DSMB) was established. Safety monitoring consisted of general assessments of participant well-being during study visits. No formal adverse event classification system was implemented, and participants were instructed to report any discomfort or concerns during the study period. Any spontaneously reported safety-relevant issues were documented by trained study personnel (ZK and JK), responsible for participant contact and data collection. Overall study oversight was provided by the principal investigators (TF and BM). No predefined safety assessment time points were implemented; monitoring occurred continuously throughout the study period. No interim analyses for safety, efficacy, or futility were conducted, as the study was exploratory in nature and all analyses were performed after completion of data collection according to the prespecified analysis plan.
